# Assessing Field Dependence–Independence Cognitive Abilities Through EEG-Based Bistable Perception Processing

**DOI:** 10.3389/fnhum.2019.00345

**Published:** 2019-10-11

**Authors:** Cristina Farmaki, Vangelis Sakkalis, Frank Loesche, Efi A. Nisiforou

**Affiliations:** ^1^Computational Bio-Medicine Laboratory, Institute of Computer Science, Foundation for Research and Technology-Hellas, Heraklion, Greece; ^2^Cognition Institute, University of Plymouth, Plymouth, United Kingdom; ^3^CogNovo, University of Plymouth, Plymouth, United Kingdom; ^4^Department of Education, University of Nicosia, Nicosia, Cyprus

**Keywords:** EEG, ERP, Field-Dependence, Field-Independence, bistable perception

## Abstract

Field Dependence–Independence (FDI) is a widely studied dimension of cognitive styles designed to measure an individual’s ability to identify embedded parts of an organized visual field as entities separate from that given field. The research aims to determine whether the brain activity features that are considered to be perceptual switching indicators could serve as robust features, differentiating Field-Dependent (FD) from Field-Independent (FI) participants. Previous research suggests that various features derived from event related potentials (ERP) and frequency features are associated with the perceptual reversal occurring during the observation of a bistable image. In this study, we combined these features in the context of a different experimental scheme using ambiguous and unambiguous stimuli during participants’ perceptual observations. We assessed the participants’ FD-I classification with the use of the Hidden Figures Test (HFT). Results show that the peak amplitude of the frontoparietal positivity, the late positive deflection in frontal and parietal areas, is higher for the FD group at specific locations of the left lobe, whereas it occurs later for the FD group at the central and occipital electrodes. Additionally, the FD group exhibits higher levels of gamma power before stimulus onset at channel TP10 and higher gamma power during reversal at the right centroparietal electrodes (T8, CP6, and TP10). The peak amplitude of the reversal positivity, the positive deflection during the reversal, is higher for the FD group at the rear right lobe (P4).

## Introduction

The Field Dependence – Independence (FDI) concept has been subject of extensive research for over 30 years and is a well-established construct for identifying individuals’ visuospatial and perceptual processing ability (Üstünel et al., 2015). Contrary to cognition, which refers to the individual’s performance capacity and is considered to be domain-specific, cognitive learning style describes relatively stable approaches of a learner toward a learning task across a range of different domains (Kahtz and Kling, 1999) After all, cognition refers to the mental ability of acquiring information, storing it and processing it to generate new knowledge. It encompasses numerous processes, from attention, memory and perception, to problem-solving and decision making (Solso et al., 2005). On the other hand, an individual’s cognitive learning style refers to the distinguishing manner to acquire, organize, manipulate, and interpret information, and addresses how these interpretations are affecting his/her actions (Hayes and Allinson, 1998). In other words, these cognitive learning styles describe a learner’s approach toward learning. Witkin et al. (1971) initially introduced the FDI construct when they developed the Hidden Figures Test (HFT). This psychological task is designed to measure an individual’s ability to identify embedded parts of an organized visual field as separate entities from that given field. According to the underlying theory, there are two different ways of processing information called *Field Dependence (FD)* and *Field Independence (FI)*. These processing styles relate to two distinct and contrasting learning and teaching methods. From this point on, the terms ‘cognitive style’ and ‘cognitive ability’ are used interchangeably and treated as synonyms.

Visual perceptiveness highlights the fundamental difference between the two types of learners. The overall visual structure of a stimulus mainly influences the pattern recognition ability of the *Field Dependent learners*. *Field Independent learners* find it easier to break up a complex figure’s visual structure and discern its distinctive pieces. More generally, FD individuals are less able to view a cue without its contextual surroundings, while FI people are better at discriminating between objects and background (Zhang, 2004).

The differences in cognitive processing underlying the performance of FD and FI individuals in visual attention tasks are not yet fully revealed. Eye tracking studies have been conducted toward this end, since eye movement patterns during visual tasks could indirectly be used to infer the participants’ mental state. Toker et al. (2013) reported the significant impact of cognitive abilities, such as perceptual speed and verbal working memory, on the user’s gaze behavior. Further studies (Nisiforou and Laghos, 2013, 2016) identified differences between the FDI cognitive abilities in terms of search tasks time completion and eye gaze patterns.

**FIGURE 1:**
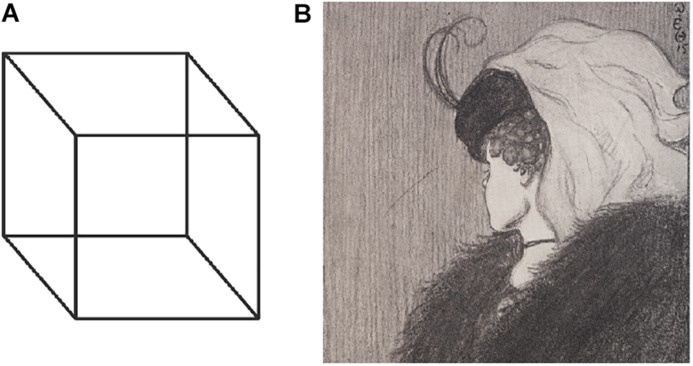
Ambiguous images: **(A)** the Necker cube, **(B)** My Wife and My Mother-in-Law.

In the current study we opted for the use of an experiment that employs ambiguous images to study the differences between FD and FI individuals. Ambiguous or bistable images, such as the Necker cube (Necker, 1832) or My Wife and My Mother-in-Law (published by William Ely Hill in an American humour magazine on 1915) are static images designed to render two different interpretations (Figure 1); although the visual stimulus does not change, the people often report spontaneous reversals between the mental states corresponding to the two interpretations. Two main approaches attempt to explain such perceptual instability phenomena, namely the bottom–up (sensory) and the top–down (cognitive) approaches. The stimulus-driven bottom–up processes suggest that neuronal responses to stimulations of the eyes are formed in the visual cortex. The top–down approach is based on the more active processes of attention, expectation, and learning (Rock et al., 1994). Although most researchers have suggested either a bottom–up or a top–down based approach, an increasing number of studies indicate that both types of perceptual processes play important roles in the explanation of the reversal phenomenon (Hochberg and Peterson, 1987; Leopold and Logothetis, 1999; Long and Toppino, 2004; Kornmeier and Bach, 2012). Intaitė et al. (2013) suggested that the perception of bistable images seems to be affected by both bottom–up and top–down processes independently, while others argue that the observed switching patterns are a result of distributed systems (Denham et al., 2018).

Recently, neuroimaging studies seem to focus on spatial aspects of brain activation in bistable perception and have, thus, used functional MRI (fMRI) and transcranial magnetic stimulation (TMS) (Knapen et al., 2011; Kleinschmidt et al., 2012; Wang et al., 2013, 2017; Weilnhammer et al., 2017). However, electroencephalography (EEG) remains a very powerful tool for capturing brain activity during this phenomenon due to its high temporal resolution, necessary for finding the precise time of the reversal event (Sakkalis, 2011a, b). The endogenous nature of the perceptual reversal phenomenon makes it challenging to establish the exact moment when the reversal occurs; thus different strategies and experimental schemata have been followed. In this study we adapt the experimental design of the ‘manual response paradigm’ (Kornmeier and Bach, 2012), where participants are asked to manually indicate the time of the reversal by pressing a button. This time point is consequently used as a time reference for the signal analysis (Başar-Eroglu et al., 1996; Isoglu-Alkaç et al., 2000; Strüber et al., 2000, 2001; Strüber and Herrmann, 2002; Mathes et al., 2006). Nevertheless, some authors (Strüber and Herrmann, 2002) assert that manual reaction times vary largely – both intra-individually and inter-individually. Therefore using stimulus onset as the time reference has been preferred in numerous works (Kornmeier and Bach, 2005; Britz et al., 2008; Pitts et al., 2008; Intaitė et al., 2010). In such case, the stimulus is presented discontinuously to the participants with short inter-stimulus intervals, during which the participants indicate the occurrence of a previous reversal by pressing a button.

The vast majority of the publications concerning bistable perception have used the same stimulus throughout their experiments. The Necker cube (Isoglu-Alkaç et al., 2000; Kornmeier and Bach, 2005; Mathes et al., 2006), Necker lattices (Britz et al., 2008; Pitts et al., 2008; Intaitė et al., 2010; Kornmeier and Bach, 2012), the Boring’s young/old woman (Boring, 1930; Kornmeier and Bach, 2009, 2014), and stroboscopic alternative motion stimuli (Başar-Eroglu et al., 1996; Strüber et al., 2000, 2001) have been the most prevalent choices. Hence, a question is raised: If different ambiguous images were used as stimuli in each trial, would the event-related potentials (ERPs) and frequency related features, indicative of perceptual reversals, still be detected? Does the effect of habituation and the expectancy of a known stimulus influence the reversal processes?

Depending on the paradigm used and the specific experiment stimulus, researchers have detected various features that indicate or are related to perception reversals. More specifically, Kornmeier and Bach (2004) used the Necker cube as a bistable stimulus and introduced an experimental paradigm that has been replicated various times after that (Kornmeier and Bach, 2005, 2006; Britz et al., 2008). The image of the Necker cube was presented to the participants in intermittent trials that lasted 800 ms, followed by a blank screen for 400 ms, during which the participants were asked to press a button whenever a perceptual reversal had occurred during the previous trial. The most prominent components differentiating the reversal from the stable condition in these publications are similar. For example, the reversal positivity appears in the time window 128–154 ms (Britz et al., 2008) and around 130 ms (Kornmeier and Bach, 2005, 2006), with stronger amplitudes of the ERPs in the reversal condition. Furthermore, the reversal negativity is similar during the time window 274–292 ms (Britz et al., 2008) and around 250 ms (Kornmeier and Bach, 2004, 2005, 2006), with ERPs more negative in the reversal condition. Finally, frontal and parietal areas exhibit a late positivity in the time window 423–471 ms (Britz et al., 2008), and around 340 ms for the frontal locations and around 470 ms for the parietal locations (Kornmeier and Bach, 2006), with more positive ERPs in the reversal condition.

Aside from poststimulus ERP components, Britz et al. (2008) also studied the relation of the prestimulus brain activity with the perceptual reversal using EEG microstates and revealed that momentary fluctuations of spontaneous brain activity before the stimulus onset influences the perceptual interpretation of bistable stimuli. An increase in low gamma power (26–40 Hz) roughly 200 ms before stimulus onset was detected by Ehm et al. (2011) during the intermittent presentation of a lattice of ambiguous Necker cubes, thus confirming the suggestions of Britz et al. (2008).

Furthermore, Isoglu-Alkaç et al. (2000) and Strüber and Herrmann (2002) have identified the reversal positivity as a P300-like component occurring about 250 ms before a key press, indicating the perceptual reversal in manual response paradigms. Isoglu-Alkaç et al. (2000) reported a decrease in the alpha power simultaneously with the P300-like reversal positivity during a manual response experiment. Similarly, Strüber and Herrmann (2002) observed a slow reduction of the alpha activity levels within 1000 ms before the button press indicating an endogenous reversal.

Moreover, instead of static bistable images, Başar-Eroglu et al. (1996) induced perceptual bistability by using a dynamic ambiguous stimulus pattern (Stroboscopic Alternative Motion: SAM). The results of their study revealed a prominent enhancement within the gamma band, more specifically at 30–50 Hz, in frontal locations, within 1000 ms before the reversal button press. In the current experiment we attempted to explore the use of various bistable figures as stimuli and to observe both ERP and frequency related previous findings combined. Consequently, we calculated ERP and frequency features using both manual response and onset paradigms. This study served a twofold purpose: (a) to confirm that previous ERP and frequency related findings indicating perceptual switching can be detected in the utilized experimental scheme, (b) to examine whether the same features could effectively differentiate FD from FI participants. To date, limited research has been performed relying on neurophysiological measures, such as EEG or fMRI, to study the individual differences of the FDI cognitive abilities and most of them were focused on the lateralization of the function in the brain and employed coherence measures (O’Connor and Shaw, 1978; Milne and Szczerbinski, 2009). This current study is the first attempt to investigate the FDI phenomenon through the neural correlates of bistable perception.

## Materials and Methods

### Participants

For this study 31 Psychology students were recruited at the University of Plymouth (United Kingdom), who received course credit as compensation. Their mean age is 21 years and 2 months (*SD* = 5.22). All participants were right-handed, with normal or corrected-to-normal vision. All people were informed about the procedure and purpose of the study. After filling in an EEG Safety Questionnaire they were asked to give their informed consent to participate in the study. The study was approved by the Ethics Committee of the Faculty of Health and Human Sciences at the University of Plymouth to be in accordance with the Declaration of Helsinki.

### Experimental Procedure

The experimental procedure consisted of three distinct tasks: (a) an ambiguous perceptual task including a training phase and a testing phase, (b) a FD-I visuospatial performance task, and (c) a creativity performance task. The experiment as a whole lasted approximately 3 h, comprising the EEG set-up procedure and the completion of the three aforementioned tasks. Throughout the ambiguous figures experiment, the participants’ brain activity was captured via a wireless head-mounted electroencephalogram (EEG) recording device combined with a synchronized eye-tracking device. Visuospatial and creative performances were assessed through the Hidden Figures Task (HFT) and the Torrance Test of Creative Thinking (TTCT) respectively. For the current paper, we focused on the neurophysiological data collected from the EEG recordings, along with the results from the HFT test. Therefore, we excluded the eye-tracking data and the results from the creativity performance task.

#### Ambiguous Perceptual Task

In the current study, we used 100 different images as stimuli: 50 ambiguous images (having two possible interpretations) for the bistable condition (condition c1), and 50 unambiguous images (having a single interpretation) for the control condition (condition c2). The images for the conditions originate from the existing collection of Snodgrass and Vanderwart (1980).

The images were scaled to the same dimension (1280 × 1024 pixels) and were presented to the participants as static black-white figures. The order of the stimuli was randomized between participants, resulting in conditions c1 and c2 alternating unpredictably throughout the experiment. The participants were asked to recognize two distinct interpretations embedded in each ambiguous figure.

**FIGURE 2:**
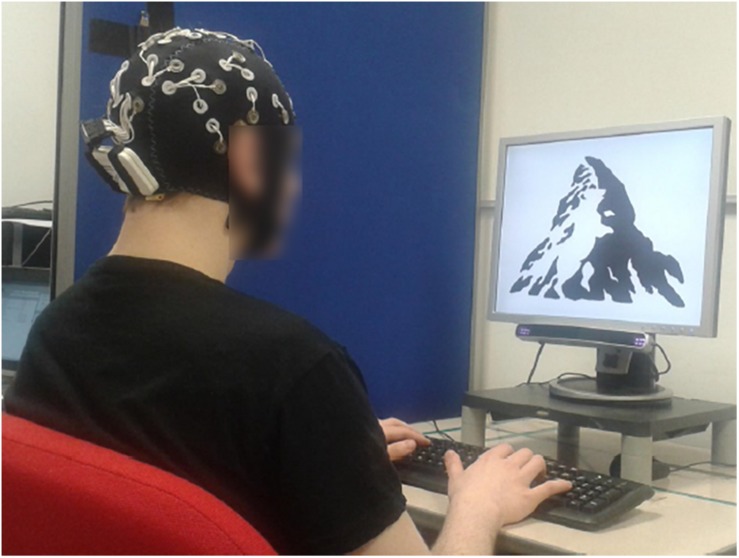
Experimental setup and exemplar of a bistable stimulus (mountain/bear) displayed on the monitor while the participant was performing the ambiguous perceptual task. The participant signed a consent for publication of this data.

All trials had a similar setup: at the beginning, participants were asked to focus their gaze at a fixation cross that appeared at the center of the screen for a randomized time between 800 and 1300 ms. Subsequently, the stimulus, either ambiguous or unambiguous, appeared. Here, participants were instructed to press a button once they perceived the initial interpretation of the image and press the same button again once the image changed into something else (a second interpretation). They could either use the ‘SPACE’ or ‘ENTER’ key to report the perceptual reversal. This setup had three possible outcomes: If the participant could perceive both interpretations, they pressed the button twice. The first button identified when participants perceived the initial interpretation, whereas the second button press identified the first reversal and ended the current trial. If the participants only reported an initial interpretation with a single button press, the experiment ended after 5000 ms. Similarly, with no initial interpretation reported through a button press, the experiment ended after 5000 ms as well. Before the start of the next trial, the image of closed eyes reminded the participants to blink between trials. Figure 2 shows a participant observing a bistable stimulus while performing the ambiguous perceptual task.

To accustom participants with the procedure, a training session was carried out, consisting of 10 trials with two pre-selected images: one ambiguous and one unambiguous. Following the training session, the testing session started. It consisted of three blocks of 100 trials using images in a randomized order, 50 belonging to the bistable condition c1, 50 to the unambiguous condition c2. After the testing session, each participant had seen 310 images in total.

#### FD-I Visuospatial Performance Task

The Field Dependence – Independence level of the participants was assessed using the Hidden Figures Test (HFT) (Ekstrom et al., 1976; Nisiforou and Laghos, 2013). The HFT consists of 32 questions/items divided equally into two parts, each one of which has a length of 12 min. Thus, the total duration for the completion of the psychometric task is 24 min. Each question/item presents five simple geometrical shapes and the participants have to determine which one of these shapes is embedded in a more complex pattern. The HFT score was calculated by taking the total correct responses minus the number of the incorrect responses, following the procedure in the manual of this test (Ekstrom et al., 1976). Reliability of the internal consistency of the Hidden Figures Test of this study was good (0.87) as measured by Cronbach’s alpha coefficient (Cronbach, 1951). Conforming to this classification framework, participants in the current study were categorized as 14 Field Dependent, 7 Field Independent learners and 10 Field Neutral learners. For the purposes of this study, only the data of the FD and FI participants were analyzed.

### EEG Recording

An mBrainTrain Smarting EEG recording device^1^ was used to capture the brain activity throughout the ambiguous perceptual task, at a sampling frequency of 500 Hz, with a 24-bit resolution. Twenty-four passive electrodes were applied to the participants’ scalp using an elastic cap, tightly fitted to their head size. The electrodes were placed according to the international 10–20 system at recording positions on the left hemisphere (Fp1, F7, FC1, C3, T7, CP1, CP5, TP9, P3, and O1), the right hemisphere (Fp2, F8, FC2, C4, T8, CP2, CP6, TP10, P4, and O2), as well as the center of the scalp (Fz, Cz, CPz, and Pz). All electrodes were applied using abrasive and conductive gels to keep the impedances below 10 kΩ. The reference electrode was positioned at FCz and a ground electrode at Fpz.

EEG data were recorded using OpenVIBE 0.18 (Renard et al., 2010) and stored using the European Data Format (EDF+), described in Kemp and Olivan (2003), as well as the General Data Format for biomedical signals (GDF), version 1.25 (Schlögl, 2006).

The design of the stimuli presentation was developed using the software OpenSesame 2.9 (Mathôt et al., 2012). In order to code timing information, OpenSesame generated events that were synchronized and combined with the incoming EEG data stream at the acquisition server level. We implemented the customized Stimulation Connection class for the mBrainTrain EEG device, which allowed a low latency inter-process communication. The EEG device received the generated events and stored them with the EEG data.

### EEG Data Analysis

The continuous EEG data of all the participants were filtered using a Hamming-windowed bandpass FIR filter of 2–65 Hz and baseline corrected, using as a baseline the time range [−1000 0] ms before stimulus onset. Afterward, for each participant and each trial, three epochs were extracted, time-locked to three different events:

• *Stimulus onset*, i.e., appearance of the image on the screen.
• *First button press*, indicating the first interpretation of the image (where no reversal takes place).
• *Second button press*, indicating the second interpretation of an image, if it exists (perceptual reversal).

**FIGURE 3:**
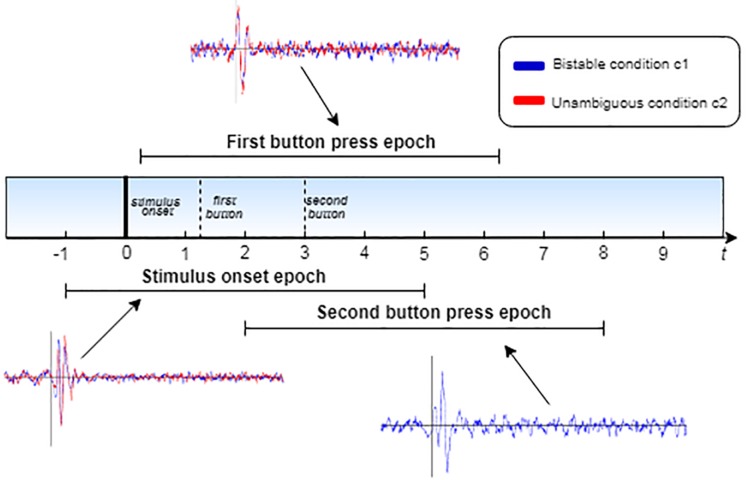
Schematic diagram of the epoch extraction process. The plots correspond to the ERPs, as calculated for each epoch, where the blue line corresponds to the bistable condition c1 and the red line to unambiguous condition c2.

Henceforth, we will refer to these different epochs as *stimulus onset epochs*, *first button epochs* and *second button epochs*. All epochs start at 1000 ms before each event and end at 5000 ms after that. In every single trial, the epochs overlap, as illustrated in Figure 3. Subsequently, the epochs of each participant were separated into two conditions (condition 1: bistable stimuli, condition 2: unambiguous stimuli). For each condition, the epochs were averaged to extract three ERPs, corresponding to the three events mentioned above, for each participant.

Afterward, we looked for findings in previous experiments (Başar-Eroglu et al., 1996; Isoglu-Alkaç et al., 2000; Britz et al., 2008; Ehm et al., 2011), in order to extract robust indicators related to perceptual switching, which could also serve as features to differentiate field-dependent from field-independent participants.

Başar-Eroglu et al. (1996), Isoglu-Alkaç et al. (2000), Strüber and Herrmann (2002), Kornmeier and Bach (2004, 2005, 2006), Britz et al. (2008), and Ehm et al. (2011) putting together the conclusions of these previous publications and after careful inspection of all the channels’ grand mean ERPs, we decided to calculate and use the following EEG features:

(1) Peak amplitude of positivity, within [100 200] ms *after stimulus onset*.
(2) Latency of positivity within [100 200] ms *after stimulus onset*.
(3) Peak amplitude of negativity, within [200 280] ms *after stimulus onset*.
(4) Latency of negativity within [200 280] ms *after stimulus onset*.
(5) Peak amplitude of frontoparietal positivity, within [280 400] ms *after stimulus onset*.
(6) Latency of frontoparietal positivity within [280 400] ms *after stimulus onset*.
(7) Peak amplitude of late positivity, within [400 600] ms *after stimulus onset*.
(8) Latency of late positivity within [400 600] ms *after stimulus onset*.
(9) Low gamma power (26–40 Hz), within [−200 −50] ms *before stimulus onset*.
(10) Peak amplitude of reversal positivity, within [−300 −200] ms *before button press*.
(11) Alpha power (8–13 Hz), within [−1000 0] ms *before button press*.
(12) Gamma power (26–60 Hz), within [−1000 0] ms *before button press*.

In order to compare between the two conditions, we calculated the features with time limits specified as ‘after stimulus onset’ or ‘before stimulus onset’ (features 1–9) for the stimulus onset epochs for both conditions. Respectively, we calculated the features with time limits specified as ‘before button press’ (features 10–12) for the first and second button epochs. Since no perception reversal occurs in the trials of the unambiguous condition c2, there is no second button press expected and no direct comparison between the conditions can be established in this case. Thus, we calculated and compared features 10–12 for both button presses, only for the bistable condition c1.

Most of the features measure ‘events of interest’ different from the baseline, which is defined as an interval [−1000 0] ms before stimulus onset. Consequently, we applied a baseline correction to all trials. Nevertheless, the time interval of feature 9, namely low gamma band power [−200 −50] ms before stimulus onset, falls within the range of the baseline interval. The effects of interest within that interval would confound with baseline correction effects. In order to overcome this complication, we calculated the last feature without the baseline correction but following the same chain of analysis described above.

The EEG data preprocessing, analysis and feature extraction used the open-source MATLAB-based toolbox EEGLAB (Delorme and Makeig, 2004), which was designed for single-trial and averaged multichannel EEG analysis. We calculated all the features referring to local peaks using the function *localpeak()* of the MATLAB-based toolbox ERPLAB (Lopez-Calderon and Luck, 2014), an extension of EEGLAB for ERP analysis.

For the frequency band power estimation we used the Welch’s overlapped segment spectral estimation method, according to which the time series is split into overlapping segments windowed by a Hamming window function. Welch’s method computes a modified periodogram for each segment, using a discrete Fourier transform, and then averages these estimates to produce the power spectral density estimate (Sakkalis, 2015). In order to facilitate comparisons with previous works, we chose to calculate the Welch’s spectral estimation either over the averaged trials or over the single trials, according to the respective publications. Specifically, Isoglu-Alkaç et al. (2000) calculated alpha band root mean square values of the average of all trials, whereas both Başar-Eroglu et al. (1996) and Ehm et al. (2011) performed spectral estimation over single trials: Ehm et al. (2011) computed the single trial short time Fourier transforms, before onset for gamma and other frequency ranges, while Başar-Eroglu et al. (1996) computed RMS amplitude values of single EEG trials for a sub-band of gamma between 30 and 50 Hz.

Therefore, for the calculation of the alpha power, we performed the Welch’s method on the time series of the averaged trials, whereas for the calculation of the low gamma power before onset and the gamma power before button press we performed the Welch’s method on each single trial and then averaged all those estimates. In all analyses we used an fft length of 500 points, a window size of 500 points and no overlap, since the time epochs of interest for each feature are 1000 ms long, or less (in the case of low gamma before onset), which is translated to epochs of 500 samples.

All the features were calculated for each one of the 24 EEG channels, as well as for the following channel groups:

• L1: Fp1, F7, FC1 (left frontal lobe)
• L2: Fp2, F8, FC2 (right frontal lobe)
• L3: TP9, CP5, CP1, P3, O1 (left rear lobe)
• L4: TP10, CP6, CP2, P4, O2 (right rear lobe)
• L5: L1 + L3 (right lobe)
• L6: L2 + L4 (left lobe)

The results form a total of 30 channels and channel groups.

### Feature Analysis

As previously mentioned, the purpose of this study is twofold, firstly to confirm and strengthen the findings of previous publications concerning the differentiation of the perceptual reversal state, compared to a non-reversal state. Secondly, we aim to investigate whether the features that characterize the perceptual reversal could also differentiate between FD and FI participants.

Therefore, in order to reveal pronounced differences between the two experimental conditions (bistable and unambiguous), we applied a paired-sample *t*-test between the same features in different conditions. There is no second button press expected for condition c2 with unambiguous images. Hence, we calculated features 1–9 around stimulus onset for both conditions, whereas we calculated features 10–12 before the first and second button press only for condition c1. In this case we applied a paired sample *t*-test between the features calculated before the first and the second button press.

Based on the score that was achieved in the visuospatial performance task, the participants were assigned to the FD and FI group. Our goal was to investigate whether the features that characterize the perceptual reversal yield from an underlying process that can discriminate the two groups of subjects. Therefore, we applied a two-sample *t*-test onto the features calculated for condition c1 only.

Table 1 summarizes all the comparisons performed for the current experiment, i.e., between bistable and unambiguous conditions, as well as between FD and FI groups, only for condition c1.

**TABLE 1:** Comparisons that were performed for the current experiment: (a) between bistable and unambiguous conditions, and (b) between FD and FI participant groups.

**Feature**	**Time window of calculated feature**	**Comparisons between conditions, *for all participants***	**Comparisons between FDI groups, *for condition c1***
Amplitude of reversal positivity	[100 200] ms	Stimulus onset epoch c1 vs. c2	Stimulus onset epoch FD vs. FI
Latency of reversal positivity	[100 200] ms.		
Amplitude of reversal negativity	[200 280] ms		
Latency of reversal negativity	[200 280] ms		
Amplitude of frontoparietal positivity	[280 400] ms		
Latency of frontoparietal positivity	[280 400] ms		
Amplitude of late positivity	[400 600] ms		
Latency of late positivity	[400 600] ms		
Low gamma power	[−200 −50] ms		
Amplitude of reversal positivity	[−300 −200] ms	1^st^ button press epoch (c1) vs.	2^nd^ button press epoch FD vs. FI
Alpha power	[−1000 0] ms	2^nd^ button press epoch (c1)	
Gamma power	[−1000 0] ms		

The Holm-Bonferroni correction method was used to control the familywise error rate introduced by multiple comparisons. All statistical analyses were performed using the Statistics and Machine Learning toolbox of MATLAB.

## Results

### Comparison Between Bistable and Unambiguous Condition

**FIGURE 4:**
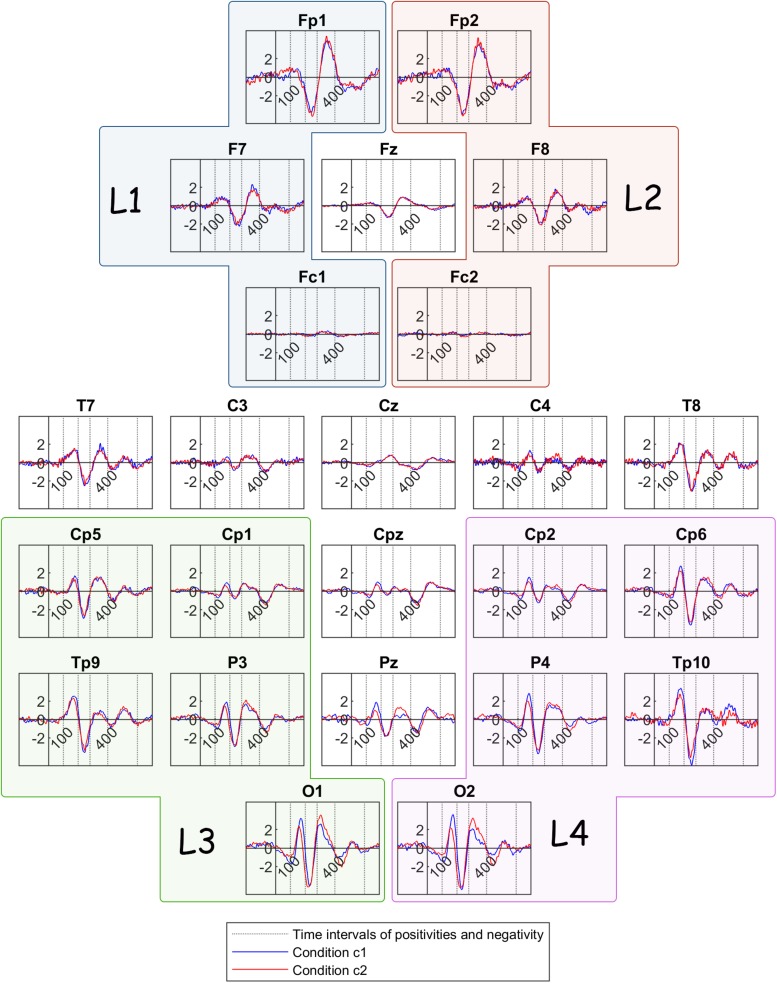
Grand mean ERP traces across all participants, for condition c1(bistable condition – blue line) and condition c2 (unambiguous condition – red line). The dotted lines show the limits of the time intervals for the features of the current study: 100, 200, 280, 400, 600 ms. L1, L2, L3, and L4 are the group channels for the features.

In order to formulate the grand mean ERPs, we averaged the ERPs with respect to the stimulus onset over participants, but separately for channels and conditions (Figure 4). The ERP components that have been identified by previous research to correlate with perceptual reversal can be detected here. More specifically, the earliest positivity component is located between 100 and 200 ms after stimulus onset and is most prominent in parietal and occipital areas. This early component is followed by a negativity component in most channels, occurring between 200 and 280 ms after onset. A frontal positivity is prominent in the time interval between 280 and 400 ms after onset, while a similar positivity component is discernible in most parietal and occipital channels during the same interval. Lastly, another positivity component can be detected in parietal areas between 400 and 600 ms after stimulus onset.

From visual inspection, it is evident that the ERP traces are similar across the two conditions, except for deflections in amplitudes and latencies mostly in parietal and occipital locations. Therefore, we performed statistical comparisons in order to reveal significant differences between the bistable and the unambiguous condition. First, we performed a comparison between the two conditions c1 and c2, for each one of the features that were calculated before stimulus onset (feature 9) and after stimulus onset (features 1–8). The statistical differences revealed between the two conditions are passing the threshold of *p* < 0.05 in all cases, where m_c__1_ is the mean value of each feature for condition c1 and m_c__2_ is the mean value of the same feature for condition c2:

• *Peak amplitude of positivity after onset*: The differences in channels Fp1, Fz, CPz, TP9, P4, O1, O2, and in channel groups L1, L3, L4, L5, L6 (for all m_c__1_ > m_c__2_) pass the threshold.
• *Latency of positivity after onset*: The differences in channels C3, CP5, CP6, TP10, Pz, P3, O1, O2, and in channel groups L4, L5, L6 (for all m_c__1_ > m_c__2_) pass the threshold.
• *Peak amplitude of negativity after onset*: The differences in channels Fp1, P3, O1 (for all m_c__1_ > m_c__2_) and Fz (m_c__1_ < m_c__2_) pass the threshold.
• *Latency of negativity after onset*: The differences in channels CP5, P4, and in channel group L3 (for all m_c__1_ > m_c__2_) pass the threshold.
• *Peak amplitude of frontoparietal positivity after onset*: The difference in channel O1 (m_c__1_ < m_c__2_) passes the threshold.
• *Latency frontoparietal positivity after onset*: The differences in channels FC1, C4 (Fc1: m_c__1_ > m_c__2_; C4: m_c__1_ < m_c__2_) pass the threshold.
• *Peak amplitude of late positivity after onset*: The differences in channels P4, O2 (for all m_c__1_ > m_c__2_) pass the threshold.
• *Latency of late positivity after onset*: The differences between the two conditions do not pass the threshold.
• *Low gamma power before onset*: The differences between the two conditions do not pass the threshold.

**FIGURE 5:**
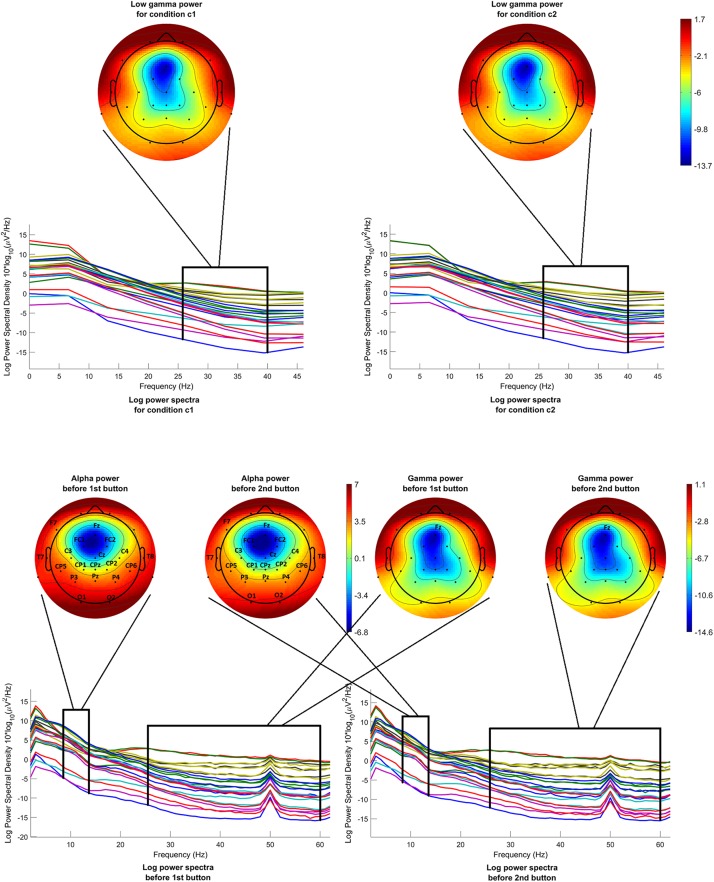
Mean log power spectra over all participants, in the interval [–200 –50]ms before onset, for each channel and for both conditions and respective scalp maps after averaging over the low gamma band (26–40 Hz) (above). Mean log power spectra over all participants, in the interval [–1000 0]ms before 1st and 2nd button, for each channel and respective scalp maps after averaging over the alpha band (8–13 Hz) (**on the left**) and over the gamma band (26–60 Hz) (**on the right**). The labels of the channels indicate the locations where the difference passed the threshold.

Low gamma power before onset was calculated and compared for both conditions c1 and c2. Figure 5 (above) shows the mean log power spectra over all participants, in the interval [−200 −50]ms before onset, for each channel in condition c1 and condition c2. In the same figure, scalp maps are drawn for both conditions, after averaging the power spectra over the desired frequency band (26–40 Hz). The scalp maps do not reveal any differences between the two conditions, as it is confirmed by the statistical analysis.

We calculated the remaining features 10–12 for the interval before the first button press, as well as for the interval before the second button press, where applicable. Below are the differences between the two intervals. Here, m_f.b_ is the mean value of each feature for the interval before first button press and m_s.b_ is the mean value of the same feature for the interval before second button press, and *p* < 0.05 in all cases.

• *Peak amplitude of reversal positivity before button press*: The differences do not pass the threshold.
• *Alpha power before button press*: The differences in channels Fz, F7, Fc1, FC2, Cz, C3, C4, T7, T8, CPz, CP1, CP2, CP5, CP6, TP9, TP10, Pz, P3, P4, O1, O2, and in channel groups L3, L4, L5, L6 (for all m_s.b_ < m_f.b_) pass the threshold.
• *Gamma power before button press*: The difference in channel Fz (m_s.b_ > m_f.b_) passes the threshold.

Alpha and gamma power were calculated and compared for condition c1, before the first button and before the second button. Figure 5 (below) shows the mean log power spectra over all participants, for each channel for the time interval [−1000 0]ms before the first button and before the second button. In the same figure, scalp maps are drawn for both cases, after averaging the power spectra over the desired frequency bands, i.e., 8–13 Hz for the alpha band and 26–60 Hz for the gamma band. The channels where the difference between the intervals before the first button and before the second button passed the threshold are highlighted with their labels. Although the scalp maps in both cases seem to be very similar, statistical analysis revealed a difference in alpha band that is generalized over almost the entire scalp. On the other hand, a difference in gamma power is revealed only in the channel Fz.

Table 2 presents the *T*-scores, *p*-values and mean values of the features for the channels where the difference between the two conditions passed the threshold. Supplementary Tables 1–3 present the *T*-scores and *p*-values of the above comparisons for all channels and features.

**TABLE 2:** Channels that showed significant difference between conditions c1 and c2, for all features.

**Features**	**Channels with significant difference between conditions c1 and c2**
Peak amplitude of positivity	**Fp1** [*t*(28) = 2.544, *p* = 0.017, m_c1_ = 3, m_c2_ = 2.35], **Fz** [*t*(28) = 2.119, *p* = 0.043, m_c1_ = 0.81, m_c2_ = 0.66]
	**CPz** [*t*(28) = 2.299, *p* = 0.029, m_c1_ = 1.79, m_c2_ = 1.56], **TP9** [*t*(28) = 2.082, *p* = 0.047, m_c1_ = 4.29, m_c2_ = 3.78]
	**P4** [*t*(28) = 2.285, *p* = 0.03, m_c1_ = 4.15, m_c2_ = 3.65], **O1** [*t*(28) = 2.401, *p* = 0.023, m_c1_ = 5.29, m_c2_ = 4.43]
	**O2** [*t*(28) = 3.169, *p* = 0.004, m_c1_ = 5.41, m_c2_ = 4.17], **L1** [*t*(28) = 2.348, *p* = 0.026, m_c1_ = 1.72, m_c2_ = 1.44]
	**L3** [*t*(28) = 2.517, *p* = 0.018, m_c1_ = 3.37, m_c2_ = 2.84], **L4** [*t*(28) = 2.878, *p* = 0.008, m_c1_ = 3.89, m_c2_ = 3.19]
	**L5** [*t*(28) = 2.765, *p* = 0.01, m_c1_ = 2.38, m_c2_ = 1.98], **L6** [*t*(28) = 2.628, *p* = 0.014, m_c1_ = 2.86, m_c2_ = 2.38]
Latency of positivity	**C3** [*t*(28) = 2.388, *p* = 0.024, m_c1_ = 166.3, m_c2_ = 150.5], **CP5** [*t*(28) = 2.451, *p* = 0.021, m_c1_ = 170, m_c2_ = 158.3]
	**CP6** [*t*(28) = 2.425, *p* = 0.022, m_c1_ = 173.9, m_c2_ = 166.1], **TP10** [*t*(28) = 2.28, *p* = 0.03, m_c1_ = 175.5, m_c2_ = 163.4]
	**Pz** [*t*(28) = 2.544, *p* = 0.017, m_c1_ = 145.2, m_c2_ = 132.3], **P3** [*t*(28) = 2.42, *p* = 0.022, m_c1_ = 163.6, m_c2_ = 152.3]
	**O1** [*t*(28) = 2.112, *p* = 0.044, m_c1_ = 166.9, m_c2_ = 155.8], **O2** [*t*(28) = 3.332, *p* = 0.002, m_c1_ = 167.6, m_c2_ = 153.4]
	**L4** [*t*(28) = 2.767, *p* = 0.01, m_c1_ = 170.4, m_c2_ = 161.1], **L5** [*t*(28) = 2.372, *p* = 0.025, m_c1_ = 165.3, m_c2_ = 156.1]
	**L6** [*t*(28) = 2.391, *p* = 0.024, m_c1_ = 170.3, m_c2_ = 161.6]
Peak amplitude of negativity	**Fp1** [*t*(28) = 2.136, *p* = 0.042, m_c1_ = −5.55, m_c2_ = −6.38], **Fz** [*t*(28) = −2.137, *p* = 0.042, m_c1_ = −1.81, m_c2_ = −1.64]
	**P3** [*t*(28) = 2.705, *p* = 0.011, m_c1_ = −3.9, m_c2_ = −4.5], **O1** [*t*(28) = 2.885, *p* = 0.007, m_c1_ = −5.75, m_c2_ = −6.71]
Latency of negativity	**CP5** [*t*(28) = 2.284, *p* = 0.03, m_c1_ = 245.4, m_c2_ = 238.8], **P4** [*t*(28) = 2.355, *p* = 0.026, m_c1_ = 243.8, m_c2_ = 235.6]
	**L3** [*t*(28) = 2.358, *p* = 0.026, m_c1_ = 241.8, m_c2_ = 235.2]
Peak amplitude of frontoparietal positivity	**O1** [*t*(28) = −2.364, *p* = 0.025, m_c__1_ = 5.08, m_c__2_ = 5.98]
Latency of frontoparietal positivity	**FC1** [*t*(28) = 2.331, *p* = 0.027, m_c1_ = 326.2, m_c2_ = 312.7]
	**C4** [*t*(28) = −2.293, *p* = 0.03, m_c1_ = 326.3, m_c2_ = 345.5]
Peak amplitude of late positivity	**P4** [*t*(28) = 2.108, *p* = 0.044, m_c1_ = 2.24, m_c2_ = 1.88]
	**O2** [*t*(28) = 2.181, *p* = 0.038, m_c1_ = 3.04, m_c2_ = 2.63]
Latency of late positivity	
Low gamma power b.s.o.	
Peak amplitude of reversal positivity b.b.p.	
Alpha power b.b.p.	**Fz** [*t*(28) = −2.605, *p* = 0.015, m_b1_ = −6.33, m_b2_ = −6.76], **F7** [t(28) = −2.606, *p* = 0.015, m_b1_ = 3.66, m_b2_ = 3.29]
	**FC1** [*t*(28) = −4.474, *p* < 0.001, m_b1_ = −6.14, m_b2_ = −6.8], **FC2** [*t*(28) = −3.344, *p* = 0.002, m_b1_ = −4.1, m_b2_ = −4.6]
	**Cz** [*t*(28) = −3.616, *p* < 0.001, m_b1_ = −3.32, m_b2_ = −3.95], **C3** [*t*(28) = −5.241, *p* < 0.001, m_b1_ = 1.54, m_b2_ = 0.51]
	**C4** [*t*(28) = −6.319, *p* < 0.001, m_b1_ = 1.55, m_b2_ = 0.69], **T7** [*t*(28) = −5.34, *p* < 0.001, m_b1_ = 4.67, m_b2_ = 3.92]
	**T8** [*t*(28) = −5.254, *p* < 0.001, m_b1_ = 4.5, m_b2_ = 3.79], **CPz** [*t*(28) = −4.966, *p* < 0.001, m_b1_ = 1.18, m_b2_ = 0.24]
	**CP1** [*t*(28) = −5.175, *p* < 0.001, m_b1_ = 1.68, m_b2_ = 0.61], **CP2** [*t*(28) = −6.88, *p* < 0.001, m_b1_ = 1.95, m_b2_ = 0.95]
	**CP5** [*t*(28) = −5.805, *p* < 0.001, m_b1_ = 4.53, m_b2_ = 3.32], **CP6** [*t*(28) = −5.776, *p* < 0.001, m_b1_ = 4.32, m_b2_ = 3.34]
	**TP9** [*t*(28) = −5.699, *p* < 0.001, m_b1_ = 5.04, m_b2_ = 4.18], **TP10** [*t*(28) = −7.053, *p* < 0.001, m_b1_ = 6.28, m_b2_ = 5.16]
	**Pz** [*t*(28) = −3.182, *p* = 0.004, m_b1_ = 3.8, m_b2_ = 3.05], **P3** [*t*(28) = −5.178, *p* < 0.001, m_b1_ = 4.71, m_b2_ = 3.48]
	**P4** [*t*(28) = −3.854, *p* < 0.001, m_b1_ = 4.27, m_b2_ = 3.37], **O1** [*t*(28) = −5.129, *p* < 0.001, m_b1_ = 6.65, m_b2_ = 5.56]
	**O2** [*t*(28) = −5.164, *p* < 0.001, m_b1_ = 6.95, m_b2_ = 5.92], **L3** [*t*(28) = −6.051, *p* < 0.001, m_b1_ = 4.58, m_b2_ = 3.48]
	**L4** [*t*(28) = −6.788, *p* < 0.001, m_b1_ = 4.76, m_b2_ = 3.75], **L5** [*t*(28) = −5.541, *p* < 0.001, m_b1_ = 3.12, m_b2_ = 2.29]
	**L6** [*t*(28) = −5.856, *p* < 0.001, m_b1_ = 3.5, m_b2_ = 2.75]
Gamma power b.b.p.	**Fz** [*t*(28) = 3.323, *p* = 0.002, m_*b*__1_ = −14.5, m_*b*__2_ = −14.3]

**T*-scores, *p-*values and mean values of each feature are given in parenthesis (m_*c1*_ is the mean value for condition c1, m_*c2*_ is the mean value for condition c2, m_*b1*_ is the mean value for first button press, and m_*b2*_ for the second button press).*

### Comparison Between Field-Dependent and Field-Independent People for Bistable Condition

**FIGURE 6:**
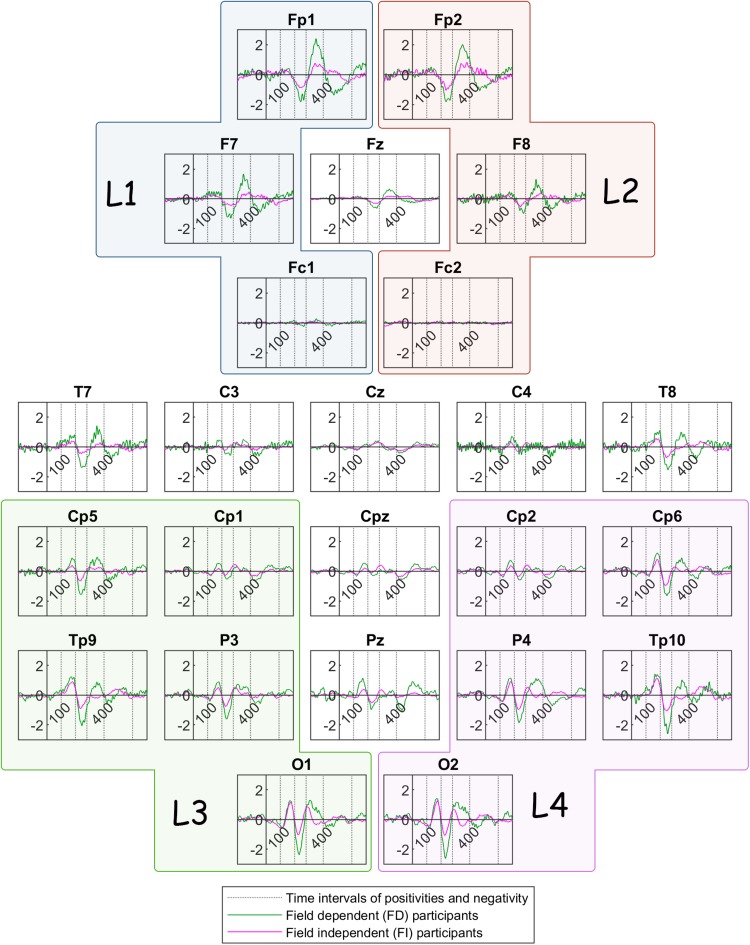
Grand mean ERP traces across Field Dependent (FD) participants (green line) and Field Independent (FI) participants (magenta line). The dotted lines show the limits of the time interval for the features of the current: 100, 200, 280, 400, 600 ms. L1, L2, L3, and L4 are the group channels for the features.

Figure 6 illustrates the grand mean ERPs for each participant group (FD and FI) in the bistable condition c1. The green color delineates traces of Field Dependent participants whereas the magenta color delineates the traces of the Field Independent participants.

Here we compare the features for condition c1 between the two groups, i.e., FD and FI participants. Similarly to the previous comparisons, m_FD_ is the mean value of each feature for the FD group, while m_FI_ is the mean value of the same feature for the FI group. All sites mentioned in the list pass the threshold of*p* < 0.05.

• *Peak amplitude of positivity after onset*: The differences in channels FC1, Cz (for all m_FD >_ m_FI_) pass the threshold.
• *Latency of positivity after onset*: The differences in channel FC1 (m_FD >_ m_FI_) pass the threshold.
• *Peak amplitude of negativity after onset*: The differences between the two groups do not pass the threshold.
• *Latency of negativity after onset*: The differences in channels FC1, C4 (for all m_FD >_ m_FI_) pass the threshold.
• *Peak amplitude of frontoparietal positivity after onset*: The differences in channels F7, T7, T8, CP5 and in channel group L1(for all m_FD >_ m_FI_) pass the threshold.
• *Latency of frontoparietal positivity after onset*: The differences in channel C3, C4, CPz, CP1, CP2, O1, O2, and in channel group L3(m_FD >_ m_FI_) pass the threshold.
• *Peak amplitude of late positivity after onset*: The differences between the two groups do not pass the threshold.
• *Latency of late positivity after onset*: The differences between the two groups do not pass the threshold.
• *Low gamma power before onset*: The differences in channel TP10 and in channel groups L1 and L4 (for all m_FD >_ m_FI_) pass the threshold.
• *Peak amplitude of reversal positivity before button press*: The difference in channel P4(m_FD >_ m_FI_) passes the threshold.
• *Alpha power before button press*: The differences between the two groups do not pass the threshold.
• *Gamma power before button press*: The differences in channels T8, CP6, TP10, and in channel groups L4, L6 (for all m_FD >_ m_FI_) pass the threshold.

In summary, the ERP of the peak amplitude of the frontoparietal positivity is higher for the FD group in the left lobe. For the same group, the peak is delayed at the central and occipital electrodes. In addition, Figure 6 reveals a trend toward a higher frontoparietal positivity for the FD group at frontal sites, as well as at certain centroparietal sites. Similarly, the negativity component seems to be ‘more negative’ for the FD group at various channels across the head. Additionally, the peak amplitude of the reversal positivity, the positive deflection during reversal, is higher for the FD group at P4.

**FIGURE 7:**
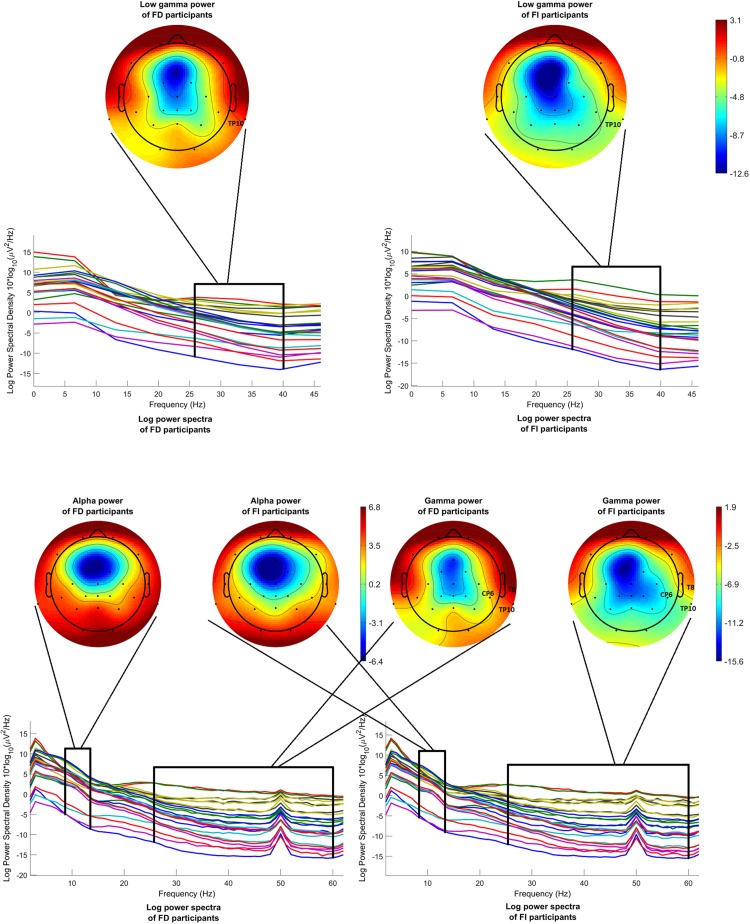
Mean log power spectra over FD and FI participants, in the interval [–200 –50]ms before onset, for each channel and for condition c1 and respective scalp maps after averaging over the low gamma band (26–40 Hz) (above). Mean log power spectra over FD and FI participants, in the interval [–1000 0]ms before 2nd button, for each channel and for condition c1 and respective scalp maps after averaging over the alpha band (8–13 Hz) (**on the left**) and over the gamma band (26–60 Hz) (**on the right**). The labels of the channels indicate the locations where the difference passed the threshold.

Figure 7 (above) shows the mean log power spectra over the FD group, on the left, and over the FI group, on the right, for each channel in condition c1. In the same figure, scalp maps are drawn for both groups, after averaging the power spectra over the desired frequency band (26–40 Hz). Although the statistical analysis revealed a difference that passed the threshold only for channel TP10, the scalp maps exhibit a tendency of the FI participants toward lower levels of low gamma power, before onset.

Figure 7 (below) illustrates the mean log power spectra over the FD group, on the left, and over the FI group, on the right, for each channel for the time interval [−1000 0]ms before the second button. In the same figure, scalp maps are drawn for both groups, after averaging the power spectra over the desired frequency bands, i.e., 8–13 Hz for the alpha band and 26–60 Hz for the gamma band. Again, the channels where the difference between the FD and FI groups passed the threshold are highlighted with their labels. The statistical analysis revealed no differences passing the threshold, regarding the alpha band, whereas the FD group exhibits higher levels of gamma power during reversal at most locations, which passed the threshold of significance at the right centroparietal electrodes (T8, CP6, and TP10).

Table 3 presents the *T*-scores, *p*-values and mean values of the features for the channels where the difference between the two FDI groups passed the threshold. Supplementary Tables 4–6 present the *T*-scores and *p*-values of the above comparisons between FDI groups, for all channels and features.

**TABLE 3:** Channels that showed significant difference between participant groups FD and FI, for all features.

**Features**	**Channels with significant difference between participants groups FD and FI**
Peak amplitude of positivity	**FC1** [*t*(19) = 2.228, *p* = 0.038, m_FD_ = 0.82, m_FI_ = 0.39]
	**Cz** [*t*(19) = 2.276, *p* = 0.035, m_FD_ = 0.98, m_FI_ = 0.35]
Latency of positivity	**FC1** [*t*(19) = 2.294, *p* = 0.033, m_FD_ = 161.1, m_FI_ = 129.1]
Peak amplitude of negativity	
Latency of negativity	**FC1** [*t*(19) = 2.613, *p* = 0.017, m_FD_ = 244.1, m_FI_ = 218.6]
	**C4** [*t*(19) = 2.12, *p* = 0.047, m_FD_ = 251.6, m_FI_ = 234]
Peak amplitude of frontoparietal positivity	**F7** [*t*(19) = 2.808, *p* = 0.011, m_FD_ = 4.41, m_FI_ = 2.68], **T7** [*t*(19) = 3.118, *p* = 0.006, m_FD_ = 4.3, m_FI_ = 1.98]
	**T8** [*t*(19) = 2.765, *p* = 0.012, m_FD_ = 3.59, m_FI_ = 1.87], **CP5** [*t*(19) = 2.334, *p* = 0.031, m_FD_ = 3.49, m_FI_ = 2.39]
	**L1** [*t*(19) = 2.872, *p* = 0.01, m_FD_ = 3.75, m_FI_ = 2.39]
Latency of frontoparietal positivity	**C3** [*t*(19) = 2.115, *p* = 0.048, m_FD_ = 337.1, m_FI_ = 307.1], **C4** [*t*(19) = 2.571, *p* = 0.019, m_FD_ = 339.3, m_FI_ = 298.6]
	**CPz** [*t*(19) = 2.534, *p* = 0.02, m_FD_ = 335, m_FI_ = 294], **CP1** [*t*(19) = 2.487, *p* = 0.022, m_FD_ = 329.4, m_FI_ = 291.1]
	**CP2** [*t*(19) = 3.374, *p* = 0.003, m_FD_ = 342.7, m_FI_ = 290.6], **O1** [*t*(19) = 2.997, *p* = 0.007, m_FD_ = 335.8, m_FI_ = 290.6]
	**O2** [*t*(19) = 2.331, *p* = 0.031, m_FD_ = 331.7, m_FI_ = 292.6], **L3** [*t*(19) = 2.791, *p* = 0.012, m_FD_ = 334.3, m_FI_ = 295.1]
Peak amplitude of late positivity	
Latency of late positivity	
Low gamma power b.s.o.	**TP10** [*t*(19) = 2.236, *p* = 0.038, m_FD_ = 0.7, m_FI_ = −3.74], **L1** [*t*(19) = 2.107, p = 0.049, m_FD_ = −2.18, m_FI_ = −5.2]
	**L4** [*t*(19) = 2.108, *p* = 0.049, m_FD_ = −2.96, m_FI_ = −5.66]
Peak amplitude of reversal positivity b.b.p.	**P4** [*t*(19) = 2.253, *p* = 0.036, m_FD_ = 1.62, m_FI_ = 0.83]
Alpha power b.b.p.	
Gamma power b.b.p.	**T8** [*t*(19) = 2.23, *p* = 0.038, m_FD_ = 0.94, m_FI_ = −3.9], **CP6** [*t*(19) = 2.255, *p* = 0.036, m_FD_ = −4.56, m_FI_ = −8.75]
	**TP10** [*t*(19) = 2.397, *p* = 0.027, m_FD_ = −0.98, m_FI_ = −6.29], **L4** [*t*(19) = 2.499, *p* = 0.022, m_FD_ = −5.1, m_FI_ = −8.78]
	**L6** [*t*(19) = 2.263, *p* = 0.036, m_FD_ = −3.99, m_FI_ = −7.26]

**T*-scores, *p-*values and mean values of each feature are given in parenthesis (m_*FD*_ is the mean value for FD group, m_*FI*_ is the mean value for FI group).*

## Discussion

In the current study, we calculated and combined various ERP-related and frequency features that have been found in previous studies to be associated with the perceptual reversal occurring during the observation of a bistable image. We aim to support the validity of such features in the context of a different experimental scheme. Most publications relevant to perceptual reversals rely on the use of a single stimulus image (usually the Necker cube, or the Boring’s old/young woman) for all their trials. In this case, the participants observe the same bistable image during every trial, thus a reversal is likely to happen, randomly, during each one of these trials. If a reversal occurs the trial is characterized as a ‘reversal’ trial. On the contrary, if no reversal takes place, the trial is characterized as a ‘stability’ one. Therefore, the same bistable stimulus can lead either to reversal or stability trials. In our study, we use 50 different bistable images and another 50 unambiguous images without perceptual reversal as a control set. Thus, various unambiguous images lead to stability trials, while various bistable stimuli lead to reversal trials (or stability ones if the participants cannot perceive the second interpretation of the image).

In addition, most experiments adopt one of the two predominant schemes, the manual response paradigm, or the onset paradigm (Kornmeier and Bach, 2012). In our setup, there was a manual indication of the time point of the reversal. Nonetheless, features were calculated both time-locked to the stimulus onset and to the reversal button press. Our findings coincide with findings mentioned in both types of previous studies.

To begin with, we found similar response intervals as previous studies, confirming a distinctive response pattern to perceptual reversals. Other studies (Kornmeier and Bach, 2004; Britz et al., 2008) developed a different experimental procedure from the one followed in the current study. Nevertheless, these authors focused on calculating prestimulus and poststimulus ERPs independent from the manual button press, which we applied to the current experimental paradigm. Their studies revealed essential ERP components that differentiated the reversal from the stable condition, such as the reversal positivity around 130 ms after stimulus onset, the reversal negativity around 280 ms after stimulus onset, and the frontoparietal positivity that occurred around 450 ms after onset. The time windows of these components coincide with the results in the current study, especially those depicted in the mean ERPs, in Figure 3.

More specifically, the early positivity component, occurring between 150 and 180 ms in the current results, has been reported in several studies using different stimuli, such as the Necker cube (Ehm et al., 2011), Necker lattices (Kornmeier and Bach, 2005, 2006; Britz et al., 2008), Boring’s old/young woman (Kornmeier and Bach, 2009, 2014), as well as binocular rivalry stimuli (Britz et al., 2011). In the current experimental scheme we use a variety of 50 different ambiguous stimuli and the result we show is for the average across all images which suggests that the measured component is related to different, if not all bistable images. Furthermore, we detect it in a number of different sites, such as the occipital area to parietal (P4), temporal-parietal (TP9), central-parietal (CPz), and even frontal (Fp1, Fz) sites. In the study by Kornmeier and Bach (2014) this component was located at occipital areas when invoked by the strictly geometrical Necker cube stimulus and it extended to occipito-temporal and parietal locations when invoked by the semantic old/young woman image. This observation could indicate that other areas besides the visual occipital area are involved in resolving the ambiguity conflict, such as temporal, specifically Fuciform Face Area (FFA), thus suggesting that the introduction of more types of stimuli involves other areas in the process of the stimulus disambiguation. This potentially contributes to the extended positivity component in our findings, as suggested by Kornmeier and Bach (2014).

The use of different ambiguous stimuli in our trials potentially influences the observed components, such as the reversal negativity component around 260 ms after stimulus onset. Our results show that the amplitude of negativity is more pronounced for the unambiguous condition, compared to the bistable condition. This finding is in contrast to the results reported by Britz et al. (2008). The exception of the Fz channel is still contradictory to their results, since that channel is located far away from the occipitoparietal channels, where the authors located the difference between conditions. Kornmeier and Bach (2004) also comment on the connection of this reversal negativity component and the perceived spatial structure of the stimulus that invoked it. The negativity component has also been detected in studies with the Necker cube (Britz et al., 2008), Schröder’s Staircase and Rubin’s vase/face stimulus (Pitts et al., 2007) and binocular rivalry stimuli (Pitts and Britz, 2011). After all, the reversal positivity component marks the initiation of the perceptual reversals and it is thought to be common for all types of bistable images, while later stages of the process depend on the spatial properties of the particular stimuli involved. In previous studies that have identified this component the perceptual reversals insinuate a change in the perceived spatial structure of the figure, i.e., a change of the participant’s viewpoint in the 3-D space, localized at occipito-parietal and temporal sites. Nevertheless, the use of the old/young woman stimulus did not invoke the negativity component (Kornmeier and Bach, 2014), which could indicate a change in semantic content of the stimulus during the reversal, instead of spatial differences. In the current study, we used a range of different stimuli and potentially each one of them could or could not invoke the negativity component at different brain areas. Depending on its spatial and semantic properties, this could result in canceling out the effect of the component and any statistically observable difference between the bistable and the unambiguous condition. Observing stimuli of various spatial and semantic characteristics could be used to establish the influence of the type of stimulus on the temporal occurrence and spatial localization of the reversal negativity component.

Following the reversal negativity after stimulus onset, visual inspection of the ERPs reveals a prominent positivity component in the time interval between 280 and 400 ms after stimulus onset, in frontal, parietal and occipital areas. Additionally, a parietal positivity component is detected between 400 and 600 ms after stimulus onset. Kornmeier and Bach (2012) refer to this component as the parietal positivity that follows the frontopolar positivity. Although they detect the frontopolar positivity (Kornmeier and Bach, 2012)only in frontal areas, it is clear from Figure 3 that in our data a positive deflection – distinct from the parietal positivity – is evident during the same interval, spanning almost the entire head. The statistical analysis places the difference of the frontoparietal positivity at the left occipital cortex (O1) and contrasts their findings (Kornmeier and Bach, 2012) because we detected a more positive deflection in the unambiguous condition than the bistable one. Furthermore, the difference in the late positivity is located in the right parietal-occipital cortex (P4, O2).

The frontopolar positivity that occurs between 280 and 400 ms after stimulus onset could be regarded as the P300 ERP component, which is related to attention and it is maximal over the central-parietal region (Polich, 2007). Meng et al. (2012) studied the relation of the ERP components and the FDI cognitive styles through a different experimental scheme. More specifically, participants in their experiment performed a stimulus matching task by categorizing two sequentially presented figures as a match (same shape) or as a conflict (different shape). Although the authors did not find a significant difference between the two groups in the P300 amplitude, they observed a tendency of larger P300 amplitudes among the FD participants relative to the FI participants, which is in line with our conclusions. The P300 component has been linked to the cognitive processes of context updating (Donchin and Coles, 1988). When Goode et al. (2002) observed similar findings in a serial-order recall task, they suggested that the larger P300 amplitude in FD participants reflects the inhibition process they must mobilize in order to change their usual global passive perception, and, instead, apply a more analytical feature-extraction approach demanded by the task. Imanaka et al. (2017) reported contrasting results in auditory and somatosensory Go/No-go paradigms, when the FI participants exhibited larger P300 amplitudes relative to their FD counterparts. This could be explained by the fact that the FD participants did not need to suppress their global-perceptual strategy during the auditory and somatosensory paradigms, thus no P300-related inhibition took place. This is indicative of how the nature of stimuli (auditory and somatosensory instead of visual) and the difference in the experimental scheme are critical for the formation of ERP components.

The increase in low gamma power (26–40 Hz) around 200 ms before stimulus onset that was detected by Ehm et al. (2011) indicated a connection between the state of the brain before stimulus onset and the perceptual interpretation of the bistable stimulus. However, the experimental scheme in this study repeatedly presents the same ambiguous stimulus, a Necker lattice and the Necker cube, respectively. Therefore, the prestimulus brain state could either invoke a perceptual reversal, or not. In the current experiment, we used both ambiguous and unambiguous stimuli in a randomized order. For the unambiguous images in condition c2, reversal is impossible irrespective of the prestimulus brain activity. Because of the randomized order, participants cannot predict the ambiguity of the next stimulus. Even if the brain state could be ‘set’ for a perceptual reversal, that reversal would not occur during the observation of an unambiguous stimulus. Across all 100 trials this could explain that, opposed to previous studies, we detect no differences between the two conditions for the low gamma power before stimulus onset.

During the expected perceptual reversal before the second button press, the differences did not pass the threshold for the peak amplitude of the reversal positivity 250 ms before the button press. This P300-like positivity has been previously identified by Isoglu-Alkaç et al. (2000), Strüber and Herrmann (2002), and Mathes et al. (2006) as an indicator of the perceptual reversal in manual response paradigms. The two buttons in the current experimental setup could be pressed with a temporal difference less than 600 ms, i.e., the sum of the late positivity component discussed earlier and the component reviewed here. Hence the movement-related potentials invoked by the first button press are likely to be superimposed on the reversal-related components and degrade their amplitude and topography characteristics (Brázdil et al., 2003; Kaòovskı et al., 2003). Consequently, our experimental scheme is not suitable for extracting information with regards to the reversal positivity before a manual response.

On the other hand, the current results suggest that the effect of the reversal on the alpha power level is not affected by the close successive button presses. As previously mentioned, Isoglu-Alkaç et al. (2000) and Strüber and Herrmann (2002) have reported a decrease in the alpha power levels within a short time window before the button press that indicates an endogenous reversal. We, on the other hand, calculated the statistical difference for feature 11 between the average alpha activity during a time interval of 1000 ms before the first and the second button press, i.e., when no reversal takes place and a reversal is expected respectively. Our results show lower alpha power during the reversal stage before pressing button two as compared to the alpha power during the stability stage before the first button press. This decrease in alpha levels could be a marker of the reversal event. The effect was generalized and spread across the entire scalp, in line with Isoglu-Alkaç et al. (2000).

Furthermore, during the same time interval, we revealed a difference between the levels of gamma band of each condition. The gamma power during bistable condition is higher in comparison with the gamma power during the unambiguous condition at the Fz channel. This site is located near F4, where Başar-Eroglu et al. (1996) identify a gamma enhancement during reversals compared to perceptual stability. They argued that the gamma band increase during bistable pattern viewing is an indication of cognitive destabilization processes underlying perceptual reversals. Similar results were reproduced in several publications after that (Strüber et al., 2000, 2001; Mathes et al., 2006).

We compare FD and FI participants using the same features previously used to compare bistable and the unambiguous conditions. We only used the features from the bistable condition c1, since we aimed to investigate whether the reversal event itself can differentiate the two groups. From a physiological perspective, various studies have revealed the relationship between the FDI cognitive ability and brain functioning. Oltman et al. (1979) reported that FD individuals demonstrate more pronounced between-hemisphere coherence and decreased hemispheric differentiation than the FI participants. FI learners exhibit better performance in the processing of specific types of information, particularly visual information (Lyons-Lawrence, 1994). Furthermore, tasks with better performances of FI learners include completion of partial occlusion images (Hodgson and Mcgonigle-Chalmers, 2011) and organization, manipulation, and restructuring of visual images (Burton et al., 1995). Considering that ‘FI individuals might recruit a strong fronto-parietal network, relating to superior feature identification and cognitive inhibition’ (Hao et al., 2013;p.7), it is probable that FI learners perform better in a visual disambiguation task. Consequently they could produce a higher reversal rate than their FD counterparts in an ambiguous stimuli experiment. The participants of the experiment in Mathes et al. (2006) volitionally speeded up or slowed down the reversal rate, or kept a passive attitude toward a Necker cube stimulus. The authors reported higher amplitude of the P300-like positivity and higher activity in the gamma sub-band 28–48 Hz during the slow down condition, compared to the speed up condition. If we assume that FD participants produce lower reversal rates than the FI participants, then the higher gamma activity during the perceptual reversal, as well as the higher amplitude of reversal positivity in the current results are explained by the findings of Mathes et al. (2006).

The differences between the two groups passed the threshold in regard to the positivity after stimulus onset (100–200 ms), the negativity after stimulus onset (200–280 ms), and the frontoparietal positivity after stimulus onset (280–400 ms). Figure 4 shows that the absolute peak amplitudes of these features are higher for the FD group, compared to the FI group.

The difference in peak amplitude and latency of the frontoparietal positivity is fascinating. FD participants exhibit a higher peak amplitude in specific sites than the FI participants. They also exhibit a longer latency than the FI participants in different locations, mainly occipital and central electrodes. The functional role of this frontoparietal positivity is not yet clear, yet some researchers have argued that it could be the indication of attentional and cognitive processes during the perceptual reversal (O’Donnell et al., 1988) or immediately after it (Isoglu-Alkaç et al., 2000). Thus, a later positivity could correlate with the poorer performance of the FD participants in attentional tasks.

## Limitations and Future Directions

As a medium to elucidate a deeper understanding of the neural underpinnings of ambiguous reversals, further experiments using stimuli of various spatial and semantic characteristics should be performed in order to establish the role of the type of the stimulus used on the occurrence and spatial localization of the reversal negativity component. Additionally, our study employed a limited number of participants from a limited demography. For a broader applicability of our findings and addressing the issue raised by Henrich et al. (2010), a replication of our experiment should incorporate more participants, from diverse backgrounds.

## Conclusion

In the current experiment bistable and unambiguous stimuli were used to investigate whether the EEG recordings of perceptual reversal events can be used to differentiate people in accordance with their FDI visuospatial ability. The findings revealed that FI learners perform better in a visual disambiguation task and produce a higher reversal rate than their FD counterparts. In addition, we observed a higher peak amplitude and latency of the frontoparietal positivity for FD participants as compared to FI participants. Furthermore, FD participants showed delayed responses at occipital and central electrodes compared to the FI participants.

The findings of the study suggest that people with higher FD-I visuospatial ability are more likely to show greater perceptual awareness and therefore experience perceptual bistability because of their robust information processing abilities. The findings can be applied by working toward better user experiences in human computer interactions, taking into account an individuals’ FD-I ability. On a fundamental level the large number of bistable stimuli employed in this experiment can be used to identify the differences in perceptual qualities of these images and how humans process them. These kinds of evidence are essential for improving brain-based research practice that will add further to the evidence and theory base of psychophysiology.

## Data Availability Statement

The datasets collected for this study can be found in the Zenodo repository at: https://doi.org/10.5281/zenodo.2589674.

## Ethics Statement

This study was carried out in accordance with the recommendations of the Ethics Committee of the Faculty of Health and Human Sciences at the University of Plymouth, with written informed consent from all subjects. All subjects gave written informed consent in accordance with the Declaration of Helsinki. The protocol was approved by the Ethics Committee of the Faculty of Health and Human Sciences at the University of Plymouth.

## Author Contributions

CF: planning and data processing, feature extraction, data interpretation, figures, initial drafting of the manuscript, structuring of the manuscript, and preparation of the manuscript. VS: planning and supervising the data analysis and interpretation, structuring of the manuscript, and critical revision of the manuscript. FL: implementation of the experiment, data collection, structuring of the manuscript, and preparation of the manuscript. EN: designing of the experiment and implementation, data collection and interpretation, theoretical background, and structuring and editing of the manuscript.

## Conflict of Interest

The authors declare that the research was conducted in the absence of any commercial or financial relationships that could be construed as a potential conflict of interest.

## Footnotes

1 https://mbraintrain.com/smarting/
