# Supplementary material for: Assessing Field Dependence–Independence Cognitive Abilities Through EEG-Based Bistable Perception Processing
Source: Front Hum Neurosci. 2019 Oct 11;13:345. doi: 10.3389/fnhum.2019.00345 (PMC6798068; doi:10.3389/fnhum.2019.00345)
Supplement: Supplementary file 1 [file Table_1.DOCX]

Supplementary Table 1 T-scores and *p* values for the comparison between conditions c1 and c2, for features 1-4 and all channels and channel groups. All features were calculated after stimulus onset and differences that pass the threshold of p<0.05 are highlighted.

| *Feature*  *Channel* | Peak amplitude of positivity | Latency of positivity | Peak amplitude of negativity | Latency of negativity |
| --- | --- | --- | --- | --- |
| Fp1 | **t(28)=2.544 , p=0.017** | t(28)=0.847 , p=0.404 | **t(28)=2.136 , p=0.042** | t(28)=0.516 , p=0.61 |
| Fp2 | t(28)=0.028 , p=0.978 | t(28)=1.249 , p=0.222 | t(28)=1.345 , p=0.189 | t(28)=1.891 , p=0.069 |
| Fz | **t(28)=2.119 , p=0.043** | t(28)=0.551 , p=0.586 | **t(28)=-2.137 , p=0.042** | t(28)=1.834 , p=0.077 |
| F7 | t(28)=2.009 , p=0.054 | t(28)=-0.407 , p=0.687 | t(28)=-0.331 , p=0.743 | t(28)=0.166 , p=0.869 |
| F8 | t(28)=1.185 , p=0.246 | t(28)=-0.672 , p=0.507 | t(28)=-0.205 , p=0.839 | t(28)=0.277 , p=0.784 |
| FC1 | t(28)=0.826 , p=0.416 | t(28)=-0.173 , p=0.864 | t(28)=-0.592 , p=0.559 | t(28)=1.359 , p=0.185 |
| FC2 | t(28)=0.834 , p=0.411 | t(28)=0.468 , p=0.644 | t(28)=1.018 , p=0.318 | t(28)=0.794 , p=0.434 |
| Cz | t(28)=-0.991 , p=0.33 | t(28)=1.619 , p=0.117 | t(28)=1.63 , p=0.114 | t(28)=2.008 , p=0.054 |
| C3 | t(28)=1.358 , p=0.185 | **t(28)=2.388 , p=0.024** | t(28)=-0.485 , p=0.631 | t(28)=1.175 , p=0.25 |
| C4 | t(28)=0.528 , p=0.602 | t(28)=0.91 , p=0.371 | t(28)=0.526 , p=0.603 | t(28)=1.005 , p=0.324 |
| T7 | t(28)=0.555 , p=0.583 | t(28)=0.819 , p=0.42 | t(28)=-0.345 , p=0.733 | t(28)=0.584 , p=0.564 |
| T8 | t(28)=0.882 , p=0.385 | t(28)=1.433 , p=0.163 | t(28)=0.345 , p=0.733 | t(28)=0.746 , p=0.462 |
| CPz | **t(28)=2.299 , p=0.029** | t(28)=1.979 , p=0.058 | t(28)=1.73 , p=0.095 | t(28)=0.143 , p=0.887 |
| CP1 | t(28)=1.411 , p=0.169 | t(28)=1.781 , p=0.086 | t(28)=1.531 , p=0.137 | t(28)=1.277 , p=0.212 |
| CP2 | t(28)=1.416 , p=0.168 | t(28)=1.918 , p=0.065 | t(28)=-0.44 , p=0.664 | t(28)=1.127 , p=0.269 |
| CP5 | t(28)=1.529 , p=0.137 | **t(28)=2.451 , p=0.021** | t(28)=0.199 , p=0.844 | **t(28)=2.284 , p=0.03** |
| CP6 | t(28)=1.265 , p=0.216 | **t(28)=2.425 , p=0.022** | t(28)=-0.764 , p=0.451 | t(28)=0.877 , p=0.388 |
| TP9 | **t(28)=2.082 , p=0.047** | t(28)=1.609 , p=0.119 | t(28)=-1.283 , p=0.21 | t(28)=1.24 , p=0.225 |
| TP10 | t(28)=1.84 , p=0.076 | **t(28)=2.28 , p=0.03** | t(28)=-1.539 , p=0.135 | t(28)=0.172 , p=0.865 |
| Pz | t(28)=2.011 , p=0.054 | **t(28)=2.544 , p=0.017** | t(28)=1.519 , p=0.14 | t(28)=2.033 , p=0.052 |
| P3 | t(28)=0.913 , p=0.369 | **t(28)=2.42 , p=0.022** | **t(28)=2.705 , p=0.011** | t(28)=0.931 , p=0.36 |
| P4 | **t(28)=2.285 , p=0.03** | t(28)=0.607 , p=0.549 | t(28)=0.153 , p=0.88 | **t(28)=2.355 , p=0.026** |
| O1 | **t(28)=2.401 , p=0.023** | **t(28)=2.112 , p=0.044** | **t(28)=2.885 , p=0.007** | t(28)=1.848 , p=0.075 |
| O2 | **t(28)=3.169 , p=0.004** | **t(28)=3.332 , p=0.002** | t(28)=1.909 , p=0.067 | t(28)=2.024 , p=0.053 |
| L1 | **t(28)=2.348 , p=0.026** | t(28)=1.247 , p=0.223 | t(28)=0.482 , p=0.634 | t(28)=1.18 , p=0.248 |
| L2 | t(28)=0.489 , p=0.628 | t(28)=0.814 , p=0.423 | t(28)=0.849 , p=0.403 | t(28)=1.272 , p=0.214 |
| L3 | **t(28)=2.517 , p=0.018** | t(28)=1.916 , p=0.066 | t(28)=1.349 , p=0.188 | **t(28)=2.358 , p=0.026** |
| L4 | **t(28)=2.878 , p=0.008** | **t(28)=2.767 , p=0.01** | t(28)=-0.739 , p=0.466 | t(28)=1.539 , p=0.135 |
| L5 (L1+L3) | **t(28)=2.765 , p=0.01** | **t(28)=2.372 , p=0.025** | t(28)=0.547 , p=0.589 | t(28)=0.997 , p=0.328 |
| L6 (L2+L4) | **t(28)=2.628 , p=0.014** | **t(28)=2.391 , p=0.024** | t(28)=-1.088 , p=0.286 | t(28)=-0.154 , p=0.878 |
