# Supplementary material for: Assessing Field Dependence–Independence Cognitive Abilities Through EEG-Based Bistable Perception Processing
Source: Front Hum Neurosci. 2019 Oct 11;13:345. doi: 10.3389/fnhum.2019.00345 (PMC6798068; doi:10.3389/fnhum.2019.00345)
Supplement: Supplementary file 2 [file Table_2.DOCX]

Supplementary Table 2 T-scores and *p* values for the comparison between conditions c1 and c2, for features 5-9 and all channels and channel groups.‘a.s.o.’ and ‘b.s.o.’ stand for ‘after stimulus onset’ and ‘before stimulus onset’, respectively. Differences that pass the threshold of p<0.05 are highlighted.

| *Feature*  *Channel* | Peak amplitude of frontoparietal positivity a.s.o. | Latency of frontoparietal positivity a.s.o. | Peak amplitude of late positivity a.s.o. | Latency of late positivity a.s.o. | Low gamma power b.s.o. |
| --- | --- | --- | --- | --- | --- |
| Fp1 | t(28)=-0.847 , p=0.404 | t(28)=0.377 , p=0.709 | t(28)=-0.32 , p=0.751 | t(28)=-0.932 , p=0.359 | t(28)=0.036 , p=0.972 |
| Fp2 | t(28)=-1.32 , p=0.198 | t(28)=-0.144 , p=0.886 | t(28)=-0.717 , p=0.479 | t(28)=-0.393 , p=0.698 | t(28)=-0.067 , p=0.947 |
| Fz | t(28)=0.192 , p=0.849 | t(28)=-0.69 , p=0.496 | t(28)=1.512 , p=0.142 | t(28)=-0.915 , p=0.368 | t(28)=0.249 , p=0.805 |
| F7 | t(28)=1.268 , p=0.215 | t(28)=0.951 , p=0.35 | t(28)=0.583 , p=0.564 | t(28)=1.432 , p=0.163 | t(28)=1.107 , p=0.278 |
| F8 | t(28)=0.156 , p=0.877 | t(28)=1.279 , p=0.211 | t(28)=-0.144 , p=0.887 | t(28)=-0.424 , p=0.675 | t(28)=-0.689 , p=0.497 |
| FC1 | t(28)=-0.199 , p=0.844 | **t(28)=2.331 , p=0.027** | t(28)=1.04 , p=0.307 | t(28)=0.801 , p=0.43 | t(28)=0.347 , p=0.731 |
| FC2 | t(28)=-1.054 , p=0.301 | t(28)=-1.925 , p=0.064 | t(28)=-0.283 , p=0.78 | t(28)=0.466 , p=0.645 | t(28)=-0.867 , p=0.393 |
| Cz | t(28)=-0.815 , p=0.422 | t(28)=-0.014 , p=0.989 | t(28)=-0.495 , p=0.624 | t(28)=1.369 , p=0.182 | t(28)=-0.826 , p=0.416 |
| C3 | t(28)=-0.038 , p=0.97 | t(28)=-1.521 , p=0.139 | t(28)=0.802 , p=0.429 | t(28)=-0.15 , p=0.882 | t(28)=0.761 , p=0.453 |
| C4 | t(28)=-1.608 , p=0.119 | **t(28)=-2.293 , p=0.03** | t(28)=-0.458 , p=0.65 | t(28)=0.91 , p=0.371 | t(28)=0.055 , p=0.956 |
| T7 | t(28)=1.01 , p=0.321 | t(28)=1.001 , p=0.326 | t(28)=0.45 , p=0.656 | t(28)=0.196 , p=0.846 | t(28)=-0.564 , p=0.577 |
| T8 | t(28)=-0.154 , p=0.879 | t(28)=-0.581 , p=0.566 | t(28)=-0.277 , p=0.784 | t(28)=-0.985 , p=0.333 | t(28)=0.166 , p=0.87 |
| CPz | t(28)=-0.976 , p=0.337 | t(28)=-1.262 , p=0.217 | t(28)=-0.588 , p=0.561 | t(28)=0.719 , p=0.478 | t(28)=-0.111 , p=0.912 |
| CP1 | t(28)=-1.009 , p=0.321 | t(28)=-0.345 , p=0.732 | t(28)=-0.577 , p=0.568 | t(28)=0.093 , p=0.926 | t(28)=-0.295 , p=0.77 |
| CP2 | t(28)=-1.484 , p=0.149 | t(28)=-1.821 , p=0.079 | t(28)=-0.042 , p=0.967 | t(28)=0.348 , p=0.731 | t(28)=0.315 , p=0.755 |
| CP5 | t(28)=-0.836 , p=0.41 | t(28)=-0.194 , p=0.848 | t(28)=0.555 , p=0.584 | t(28)=-1.555 , p=0.131 | t(28)=-1.224 , p=0.231 |
| CP6 | t(28)=-1.671 , p=0.106 | t(28)=0.924 , p=0.363 | t(28)=0.447 , p=0.658 | t(28)=-0.397 , p=0.694 | t(28)=1.161 , p=0.256 |
| TP9 | t(28)=-0.378 , p=0.708 | t(28)=0.751 , p=0.459 | t(28)=0.263 , p=0.794 | t(28)=-1.376 , p=0.18 | t(28)=0.513 , p=0.612 |
| TP10 | t(28)=-0.752 , p=0.458 | t(28)=0.725 , p=0.475 | t(28)=-0.751 , p=0.459 | t(28)=-1.341 , p=0.191 | t(28)=1.471 , p=0.152 |
| Pz | t(28)=-1.68 , p=0.104 | t(28)=-0.75 , p=0.46 | t(28)=0.612 , p=0.546 | t(28)=0.32 , p=0.751 | t(28)=-0.076 , p=0.94 |
| P3 | t(28)=-1.944 , p=0.062 | t(28)=-0.423 , p=0.675 | t(28)=-0.421 , p=0.677 | t(28)=-0.565 , p=0.576 | t(28)=-0.607 , p=0.549 |
| P4 | t(28)=-0.95 , p=0.35 | t(28)=-0.118 , p=0.907 | **t(28)=2.108 , p=0.044** | t(28)=-0.467 , p=0.644 | t(28)=-0.059 , p=0.954 |
| O1 | **t(28)=-2.364 , p=0.025** | t(28)=-0.557 , p=0.582 | t(28)=0.892 , p=0.38 | t(28)=-1.678 , p=0.104 | t(28)=0.29 , p=0.774 |
| O2 | t(28)=-1.999 , p=0.055 | t(28)=-1.394 , p=0.174 | **t(28)=2.181 , p=0.038** | t(28)=-1.902 , p=0.068 | t(28)=0.21 , p=0.835 |
| L1 | t(28)=0.494 , p=0.625 | t(28)=-0.196 , p=0.846 | t(28)=0.104 , p=0.918 | t(28)=0.856 , p=0.399 | t(28)=0.898 , p=0.377 |
| L2 | t(28)=-1.269 , p=0.215 | t(28)=0.195 , p=0.847 | t(28)=-0.467 , p=0.644 | t(28)=-0.887 , p=0.383 | t(28)=-0.68 , p=0.502 |
| L3 | t(28)=-1.457 , p=0.156 | t(28)=-0.342 , p=0.735 | t(28)=-0.111 , p=0.913 | t(28)=-1.978 , p=0.058 | t(28)=-0.302 , p=0.765 |
| L4 | t(28)=-1.644 , p=0.111 | t(28)=0.511 , p=0.613 | t(28)=0.292 , p=0.772 | t(28)=-1.762 , p=0.089 | t(28)=0.967 , p=0.342 |
| L5 (L1+L3) | t(28)=-0.679 , p=0.503 | t(28)=0.06 , p=0.953 | t(28)=0.646 , p=0.524 | t(28)=-1.041 , p=0.307 | t(28)=0.202 , p=0.841 |
| L6 (L2+L4) | t(28)=-1.314 , p=0.2 | t(28)=-0.066 , p=0.948 | t(28)=-0.677 , p=0.504 | t(28)=-0.994 , p=0.329 | t(28)=0.269 , p=0.79 |
