# Supplementary material for: Assessing Field Dependence–Independence Cognitive Abilities Through EEG-Based Bistable Perception Processing
Source: Front Hum Neurosci. 2019 Oct 11;13:345. doi: 10.3389/fnhum.2019.00345 (PMC6798068; doi:10.3389/fnhum.2019.00345)
Supplement: Supplementary file 3 [file Table_3.DOCX]

Supplementary Τable 3 T-scores and p values for the comparison between intervals before first button press and before second button press for condition c1, for features 10-12 and all channels and channel groups. All features were calculated before button press and differences that pass the threshold of p<0.05 are highlighted.

| *Feature*  *Channel* | Peak amplitude of reversal positivity | Alpha power | Gamma power |
| --- | --- | --- | --- |
| Fp1 | t(28)=-0.777 , p=0.443 | t(28)=0.411 , p=0.684 | t(28)=0.839 , p=0.408 |
| Fp2 | t(28)=-0.688 , p=0.497 | t(28)=0.679 , p=0.503 | t(28)=1.746 , p=0.092 |
| Fz | t(28)=-0.558 , p=0.581 | **t(28)=-2.605 , p=0.015** | **t(28)=3.323 , p=0.002** |
| F7 | t(28)=0.923 , p=0.364 | **t(28)=-2.606 , p=0.015** | t(28)=0.711 , p=0.483 |
| F8 | t(28)=-0.801 , p=0.43 | t(28)=-1.27 , p=0.215 | t(28)=0.837 , p=0.41 |
| FC1 | t(28)=0.912 , p=0.369 | **t(28)=-4.474 , p<0.001** | t(28)=-1.293 , p=0.206 |
| FC2 | t(28)=-1.367 , p=0.182 | **t(28)=-3.344 , p=0.002** | t(28)=-0.798 , p=0.432 |
| Cz | t(28)=-0.613 , p=0.545 | **t(28)=-3.616 , p<0.001** | t(28)=-0.465 , p=0.645 |
| C3 | t(28)=-0.085 , p=0.933 | **t(28)=-5.241 , p<0.001** | t(28)=-1.253 , p=0.22 |
| C4 | t(28)=1.315 , p=0.199 | **t(28)=-6.319 , p<0.001** | t(28)=-1.298 , p=0.205 |
| T7 | t(28)=1.488 , p=0.148 | **t(28)=-5.34 , p<0.001** | t(28)=-0.22 , p=0.828 |
| T8 | t(28)=0.691 , p=0.495 | **t(28)=-5.254 , p<0.001** | t(28)=0.023 , p=0.982 |
| CPz | t(28)=0.529 , p=0.601 | **t(28)=-4.966 , p<0.001** | t(28)=0.005 , p=0.996 |
| CP1 | t(28)=-0.129 , p=0.899 | **t(28)=-5.175 , p<0.001** | t(28)=-1.248 , p=0.222 |
| CP2 | t(28)=0.736 , p=0.468 | **t(28)=-6.88 , p<0.001** | t(28)=-0.07 , p=0.945 |
| CP5 | t(28)=-0.386 , p=0.703 | **t(28)=-5.805 , p<0.001** | t(28)=-0.461 , p=0.649 |
| CP6 | t(28)=0.499 , p=0.622 | **t(28)=-5.776 , p<0.001** | t(28)=-0.334 , p=0.741 |
| TP9 | t(28)=0.039 , p=0.969 | **t(28)=-5.699 , p<0.001** | t(28)=-0.728 , p=0.472 |
| TP10 | t(28)=0.717 , p=0.479 | **t(28)=-7.053 , p<0.001** | t(28)=-0.708 , p=0.485 |
| Pz | t(28)=1.055 , p=0.3 | **t(28)=-3.182 , p=0.004** | t(28)=-0.071 , p=0.944 |
| P3 | t(28)=-0.735 , p=0.469 | **t(28)=-5.178 , p<0.001** | t(28)=-0.979 , p=0.336 |
| P4 | t(28)=0.446 , p=0.659 | **t(28)=-3.854 , p<0.001** | t(28)=0.621 , p=0.54 |
| O1 | t(28)=-0.569 , p=0.574 | **t(28)=-5.129 , p<0.001** | t(28)=0.138 , p=0.891 |
| O2 | t(28)=0.568 , p=0.575 | **t(28)=-5.164 , p<0.001** | t(28)=0.177 , p=0.861 |
| L1 | t(28)=-0.758 , p=0.455 | t(28)=-2.029 , p=0.052 | t(28)=0.037 , p=0.971 |
| L2 | t(28)=-1.017 , p=0.318 | t(28)=-1.642 , p=0.112 | t(28)=0.607 , p=0.548 |
| L3 | t(28)=-0.209 , p=0.836 | **t(28)=-6.051 , p<0.001** | t(28)=-0.707 , p=0.485 |
| L4 | t(28)=0.157 , p=0.877 | **t(28)=-6.788 , p<0.001** | t(28)=-0.196 , p=0.846 |
| L5 (L1+L3) | t(28)=-1.513 , p=0.141 | **t(28)=-5.541 , p<0.001** | t(28)=-0.506 , p=0.617 |
| L6 (L2+L4) | t(28)=-0.38 , p=0.707 | **t(28)=-5.856 , p<0.001** | t(28)=-0.138 , p=0.891 |
