# Supplementary material for: Assessing Field Dependence–Independence Cognitive Abilities Through EEG-Based Bistable Perception Processing
Source: Front Hum Neurosci. 2019 Oct 11;13:345. doi: 10.3389/fnhum.2019.00345 (PMC6798068; doi:10.3389/fnhum.2019.00345)
Supplement: Supplementary file 4 [file Table_4.DOCX]

Supplementary Table 4 T-scores and *p* values for the comparison between participant groups FD and FI, in condition c1, for features 1-4 and all channels and channel groups. All features were calculated after stimulus onset and differences that pass the threshold of p<0.05 are highlighted.

| *Feature*  *Channel* | Peak amplitude of positivity | Latency of positivity | Peak amplitude of negativity | Latency of negativity |
| --- | --- | --- | --- | --- |
| Fp1 | t(19)=1.077 , p=0.295 | t(19)=0.597 , p=0.557 | t(19)=-0.462 , p=0.65 | t(19)=0.75 , p=0.463 |
| Fp2 | t(19)=0.794 , p=0.437 | t(19)=-0.503 , p=0.621 | t(19)=-0.155 , p=0.878 | t(19)=0.482 , p=0.636 |
| Fz | t(19)=0.842 , p=0.41 | t(19)=0.383 , p=0.706 | t(19)=-0.622 , p=0.541 | t(19)=0.733 , p=0.473 |
| F7 | t(19)=1.341 , p=0.196 | t(19)=0.492 , p=0.628 | t(19)=-1.803 , p=0.087 | t(19)=1.201 , p=0.244 |
| F8 | t(19)=0.697 , p=0.494 | t(19)=-0.035 , p=0.972 | t(19)=0.028 , p=0.978 | t(19)=0.1 , p=0.922 |
| FC1 | **t(19)=2.228 , p=0.038** | **t(19)=2.294 , p=0.033** | t(19)=-1.769 , p=0.093 | **t(19)=2.613 , p=0.017** |
| FC2 | t(19)=0.566 , p=0.578 | t(19)=-0.105 , p=0.918 | t(19)=-0.98 , p=0.339 | t(19)=0.716 , p=0.483 |
| Cz | **t(19)=2.276 , p=0.035** | t(19)=0.559 , p=0.582 | t(19)=-0.836 , p=0.413 | t(19)=1.571 , p=0.133 |
| C3 | t(19)=1.644 , p=0.117 | t(19)=1.189 , p=0.249 | t(19)=-1.689 , p=0.108 | t(19)=1.615 , p=0.123 |
| C4 | t(19)=0.718 , p=0.482 | t(19)=-0.7 , p=0.493 | t(19)=-0.161 , p=0.873 | **t(19)=2.12 , p=0.047** |
| T7 | t(19)=1.054 , p=0.305 | t(19)=0.647 , p=0.525 | t(19)=-1.533 , p=0.142 | t(19)=0.421 , p=0.678 |
| T8 | t(19)=0.65 , p=0.524 | t(19)=-0.028 , p=0.978 | t(19)=-0.566 , p=0.578 | t(19)=1.97 , p=0.064 |
| CPz | t(19)=0.987 , p=0.336 | t(19)=-0.055 , p=0.957 | t(19)=0.014 , p=0.989 | t(19)=1.397 , p=0.178 |
| CP1 | t(19)=1.025 , p=0.318 | t(19)=0.185 , p=0.855 | t(19)=-0.089 , p=0.93 | t(19)=0.945 , p=0.357 |
| CP2 | t(19)=0.143 , p=0.888 | t(19)=-0.163 , p=0.872 | t(19)=0.856 , p=0.403 | t(19)=1.106 , p=0.283 |
| CP5 | t(19)=0.769 , p=0.451 | t(19)=0.517 , p=0.611 | t(19)=-0.691 , p=0.498 | t(19)=1.638 , p=0.118 |
| CP6 | t(19)=-0.312 , p=0.758 | t(19)=-0.323 , p=0.75 | t(19)=0.609 , p=0.55 | t(19)=2.032 , p=0.056 |
| TP9 | t(19)=-0.627 , p=0.538 | t(19)=-0.263 , p=0.796 | t(19)=-0.457 , p=0.653 | t(19)=-0.116 , p=0.909 |
| TP10 | t(19)=-0.607 , p=0.551 | t(19)=-0.034 , p=0.974 | t(19)=-0.516 , p=0.612 | t(19)=0.578 , p=0.57 |
| Pz | t(19)=0.401 , p=0.693 | t(19)=-0.451 , p=0.657 | t(19)=0.404 , p=0.691 | t(19)=-0.297 , p=0.77 |
| P3 | t(19)=-0.028 , p=0.978 | t(19)=-0.772 , p=0.45 | t(19)=0.094 , p=0.926 | t(19)=-0.315 , p=0.756 |
| P4 | t(19)=-0.742 , p=0.467 | t(19)=-1.659 , p=0.113 | t(19)=0.225 , p=0.825 | t(19)=1.632 , p=0.119 |
| O1 | t(19)=-0.842 , p=0.41 | t(19)=-0.644 , p=0.527 | t(19)=-0.302 , p=0.766 | t(19)=0.909 , p=0.375 |
| O2 | t(19)=-0.961 , p=0.349 | t(19)=-0.745 , p=0.466 | t(19)=-0.366 , p=0.718 | t(19)=0.851 , p=0.405 |
| L1 | t(19)=1.297 , p=0.21 | t(19)=0.576 , p=0.571 | t(19)=-1.204 , p=0.244 | t(19)=1.95 , p=0.066 |
| L2 | t(19)=0.605 , p=0.553 | t(19)=0.29 , p=0.775 | t(19)=-0.103 , p=0.919 | t(19)=0.107 , p=0.916 |
| L3 | t(19)=-0.223 , p=0.826 | t(19)=0.225 , p=0.824 | t(19)=-0.397 , p=0.696 | t(19)=1.376 , p=0.185 |
| L4 | t(19)=-0.886 , p=0.387 | t(19)=-0.372 , p=0.714 | t(19)=0.067 , p=0.947 | t(19)=1.115 , p=0.279 |
| L5 (L1+L3) | t(19)=0.271 , p=0.789 | t(19)=0.215 , p=0.832 | t(19)=-0.775 , p=0.448 | t(19)=1.132 , p=0.272 |
| L6 (L2+L4) | t(19)=-0.361 , p=0.722 | t(19)=-0.253 , p=0.803 | t(19)=-0.048 , p=0.962 | t(19)=0.924 , p=0.367 |
