# Supplementary material for: Assessing Field Dependence–Independence Cognitive Abilities Through EEG-Based Bistable Perception Processing
Source: Front Hum Neurosci. 2019 Oct 11;13:345. doi: 10.3389/fnhum.2019.00345 (PMC6798068; doi:10.3389/fnhum.2019.00345)
Supplement: Supplementary file 5 [file Table_5.DOCX]

Supplementary Table 5 T-scores and *p* values for the comparison between participant groups FD and FI, in condition c1, for features 5-8 and all channels and channel groups. All features were calculated after stimulus onset and differences that pass the threshold of p<0.05 are highlighted.

| *Feature*  *Channel* | Peak amplitude of frontoparietal positivity | Latency of frontoparietal positivity | Peak amplitude of late positivity | Latency of late positivity |
| --- | --- | --- | --- | --- |
| Fp1 | t(19)=2.085 , p=0.051 | t(19)=-0.56 , p=0.582 | t(19)=0.709 , p=0.487 | t(19)=0.247 , p=0.807 |
| Fp2 | t(19)=0.79 , p=0.439 | t(19)=-0.822 , p=0.421 | t(19)=-0.658 , p=0.518 | t(19)=-0.021 , p=0.983 |
| Fz | t(19)=1.702 , p=0.105 | t(19)=-0.515 , p=0.612 | t(19)=-1.149 , p=0.265 | t(19)=-1.131 , p=0.272 |
| F7 | **t(19)=2.808 , p=0.011** | t(19)=-1.374 , p=0.185 | t(19)=-1.626 , p=0.12 | t(19)=0.005 , p=0.996 |
| F8 | t(19)=1.771 , p=0.093 | t(19)=-0.431 , p=0.672 | t(19)=-0.243 , p=0.81 | t(19)=0.224 , p=0.825 |
| FC1 | t(19)=1.939 , p=0.068 | t(19)=0.912 , p=0.373 | t(19)=1.475 , p=0.157 | t(19)=-0.133 , p=0.895 |
| FC2 | t(19)=0.681 , p=0.504 | t(19)=0.193 , p=0.849 | t(19)=1.881 , p=0.075 | t(19)=0.611 , p=0.548 |
| Cz | t(19)=-0.48 , p=0.637 | t(19)=1.975 , p=0.063 | t(19)=1.11 , p=0.281 | t(19)=1.255 , p=0.225 |
| C3 | t(19)=1.307 , p=0.207 | **t(19)=2.115 , p=0.048** | t(19)=1.283 , p=0.215 | t(19)=0.027 , p=0.979 |
| C4 | t(19)=0.941 , p=0.359 | **t(19)=2.571 , p=0.019** | t(19)=1.076 , p=0.295 | t(19)=-1.252 , p=0.226 |
| T7 | **t(19)=3.118 , p=0.006** | t(19)=1.795 , p=0.089 | t(19)=-0.232 , p=0.819 | t(19)=0.38 , p=0.708 |
| T8 | **t(19)=2.765 , p=0.012** | t(19)=-1.327 , p=0.2 | t(19)=-0.036 , p=0.972 | t(19)=0.921 , p=0.369 |
| CPz | t(19)=-0.781 , p=0.445 | **t(19)=2.534 , p=0.02** | t(19)=1.083 , p=0.292 | t(19)=-0.837 , p=0.413 |
| CP1 | t(19)=-0.702 , p=0.491 | **t(19)=2.487 , p=0.022** | t(19)=1.276 , p=0.217 | t(19)=-0.881 , p=0.389 |
| CP2 | t(19)=-0.524 , p=0.606 | **t(19)=3.374 , p=0.003** | t(19)=0.605 , p=0.552 | t(19)=-0.758 , p=0.458 |
| CP5 | **t(19)=2.334 , p=0.031** | t(19)=1.068 , p=0.299 | t(19)=0.875 , p=0.393 | t(19)=0.19 , p=0.851 |
| CP6 | t(19)=1.26 , p=0.223 | t(19)=1.464 , p=0.159 | t(19)=0.269 , p=0.791 | t(19)=-1.752 , p=0.096 |
| TP9 | t(19)=2.037 , p=0.056 | t(19)=1.494 , p=0.151 | t(19)=-0.444 , p=0.662 | t(19)=0.068 , p=0.946 |
| TP10 | t(19)=2.012 , p=0.059 | t(19)=0.385 , p=0.704 | t(19)=0.395 , p=0.697 | t(19)=0.18 , p=0.859 |
| Pz | t(19)=0.206 , p=0.839 | t(19)=0.048 , p=0.962 | t(19)=0.955 , p=0.352 | t(19)=-0.323 , p=0.75 |
| P3 | t(19)=0.365 , p=0.719 | t(19)=0.464 , p=0.648 | t(19)=0.385 , p=0.704 | t(19)=-0.894 , p=0.383 |
| P4 | t(19)=1.86 , p=0.078 | t(19)=1.445 , p=0.165 | t(19)=0.345 , p=0.734 | t(19)=-1.41 , p=0.175 |
| O1 | t(19)=0.618 , p=0.544 | **t(19)=2.997 , p=0.007** | t(19)=0.574 , p=0.573 | t(19)=-1.105 , p=0.283 |
| O2 | t(19)=1.363 , p=0.189 | **t(19)=2.331 , p=0.031** | t(19)=0.666 , p=0.513 | t(19)=-0.859 , p=0.401 |
| L1 | **t(19)=2.872 , p=0.01** | t(19)=0.136 , p=0.893 | t(19)=0.144 , p=0.887 | t(19)=-0.419 , p=0.68 |
| L2 | t(19)=1.309 , p=0.206 | t(19)=-1.532 , p=0.142 | t(19)=-1.043 , p=0.31 | t(19)=0.498 , p=0.624 |
| L3 | t(19)=1.127 , p=0.274 | **t(19)=2.791 , p=0.012** | t(19)=0.485 , p=0.633 | t(19)=-0.832 , p=0.416 |
| L4 | t(19)=1.663 , p=0.113 | t(19)=1.74 , p=0.098 | t(19)=-0.114 , p=0.911 | t(19)=-1.971 , p=0.064 |
| L5 (L1+L3) | t(19)=1.622 , p=0.121 | t(19)=2.002 , p=0.06 | t(19)=-0.163 , p=0.873 | t(19)=-0.171 , p=0.866 |
| L6 (L2+L4) | t(19)=2.035 , p=0.056 | t(19)=1.104 , p=0.284 | t(19)=-0.175 , p=0.863 | t(19)=-1.124 , p=0.275 |
