# Supplementary material for: Assessing Field Dependence–Independence Cognitive Abilities Through EEG-Based Bistable Perception Processing
Source: Front Hum Neurosci. 2019 Oct 11;13:345. doi: 10.3389/fnhum.2019.00345 (PMC6798068; doi:10.3389/fnhum.2019.00345)
Supplement: Supplementary file 6 [file Table_6.DOCX]

Supplementary Table 6 T-scores and *p* values for the comparison between participant groups FD and FI, in condition c1, for features 9-12 and all channels and channel groups. ‘b.s.o.’ and ‘b.b.p.’ stand for ‘before stimulus onset’ and ‘before button press’, respectively. Differences that pass the threshold of p<0.05 are highlighted.

| *Feature*  *Channel* | Low gamma power b.s.o. | Peak amplitude of reversal positivity b.b.p. | Alpha power b.b.p. | Gamma power b.b.p. |
| --- | --- | --- | --- | --- |
| Fp1 | t(19)=1.758 , p=0.095 | t(19)=0.861 , p=0.4 | t(19)=1.049 , p=0.307 | t(19)=0.847 , p=0.408 |
| Fp2 | t(19)=0.163 , p=0.873 | t(19)=0.263 , p=0.795 | t(19)=0.1 , p=0.921 | t(19)=-0.39 , p=0.701 |
| Fz | t(19)=1.364 , p=0.189 | t(19)=0.834 , p=0.415 | t(19)=0.525 , p=0.606 | t(19)=1.092 , p=0.289 |
| F7 | t(19)=1.152 , p=0.263 | t(19)=0.863 , p=0.399 | t(19)=0.597 , p=0.557 | t(19)=1.135 , p=0.27 |
| F8 | t(19)=0.845 , p=0.409 | t(19)=0.937 , p=0.36 | t(19)=1.632 , p=0.119 | t(19)=1.527 , p=0.143 |
| FC1 | t(19)=2.033 , p=0.056 | t(19)=1.399 , p=0.178 | t(19)=1.119 , p=0.277 | t(19)=1.982 , p=0.062 |
| FC2 | t(19)=-0.036 , p=0.972 | t(19)=1.937 , p=0.068 | t(19)=-0.055 , p=0.956 | t(19)=0.352 , p=0.728 |
| Cz | t(19)=0.707 , p=0.488 | t(19)=1.147 , p=0.265 | t(19)=1.096 , p=0.287 | t(19)=0.557 , p=0.584 |
| C3 | t(19)=1.347 , p=0.194 | t(19)=0.918 , p=0.37 | t(19)=1.348 , p=0.193 | t(19)=1.245 , p=0.228 |
| C4 | t(19)=1.015 , p=0.323 | t(19)=0.967 , p=0.346 | t(19)=1.098 , p=0.286 | t(19)=1.35 , p=0.193 |
| T7 | t(19)=1.834 , p=0.082 | t(19)=0.449 , p=0.658 | t(19)=0.65 , p=0.524 | t(19)=1.83 , p=0.083 |
| T8 | t(19)=2.017 , p=0.058 | t(19)=1.86 , p=0.078 | t(19)=1.346 , p=0.194 | **t(19)=2.23 , p=0.038** |
| CPz | t(19)=1.433 , p=0.168 | t(19)=0.734 , p=0.472 | t(19)=1.778 , p=0.091 | t(19)=2.092 , p=0.05 |
| CP1 | t(19)=1.579 , p=0.131 | t(19)=1.128 , p=0.273 | t(19)=1.536 , p=0.141 | t(19)=2.03 , p=0.057 |
| CP2 | t(19)=1.377 , p=0.184 | t(19)=0.58 , p=0.569 | t(19)=1.316 , p=0.204 | t(19)=2.048 , p=0.055 |
| CP5 | t(19)=1.318 , p=0.203 | t(19)=1.039 , p=0.312 | t(19)=1.183 , p=0.252 | t(19)=1.372 , p=0.186 |
| CP6 | t(19)=1.9 , p=0.073 | t(19)=1.389 , p=0.181 | t(19)=1.394 , p=0.179 | **t(19)=2.255 , p=0.036** |
| TP9 | t(19)=0.616 , p=0.546 | t(19)=-0.814 , p=0.426 | t(19)=0.503 , p=0.621 | t(19)=0.817 , p=0.424 |
| TP10 | **t(19)=2.236 , p=0.038** | t(19)=1.034 , p=0.314 | t(19)=1.893 , p=0.074 | **t(19)=2.397 , p=0.027** |
| Pz | t(19)=1.65 , p=0.115 | t(19)=-0.457 , p=0.653 | t(19)=1.57 , p=0.133 | t(19)=1.719 , p=0.102 |
| P3 | t(19)=1.796 , p=0.088 | t(19)=0.716 , p=0.483 | t(19)=1.37 , p=0.187 | t(19)=2.012 , p=0.059 |
| P4 | t(19)=1.208 , p=0.242 | **t(19)=2.253 , p=0.036** | t(19)=0.273 , p=0.788 | t(19)=2.043 , p=0.055 |
| O1 | t(19)=1.037 , p=0.313 | t(19)=1.492 , p=0.152 | t(19)=0.654 , p=0.521 | t(19)=1.228 , p=0.234 |
| O2 | t(19)=1.839 , p=0.082 | t(19)=1.405 , p=0.176 | t(19)=1.313 , p=0.205 | t(19)=2.007 , p=0.059 |
| L1 | **t(19)=2.107 , p=0.049** | t(19)=1.044 , p=0.31 | t(19)=1.051 , p=0.306 | t(19)=1.629 , p=0.12 |
| L2 | t(19)=0.526 , p=0.605 | t(19)=0.511 , p=0.615 | t(19)=0.285 , p=0.778 | t(19)=0.838 , p=0.412 |
| L3 | t(19)=1.461 , p=0.16 | t(19)=0.725 , p=0.478 | t(19)=1.18 , p=0.253 | t(19)=1.724 , p=0.101 |
| L4 | **t(19)=2.108 , p=0.049** | t(19)=1.292 , p=0.212 | t(19)=1.541 , p=0.14 | **t(19)=2.499 , p=0.022** |
| L5 (L1+L3) | t(19)=1.841 , p=0.081 | t(19)=0.125 , p=0.902 | t(19)=1.135 , p=0.271 | t(19)=1.754 , p=0.096 |
| L6 (L2+L4) | t(19)=1.817 , p=0.085 | t(19)=1.853 , p=0.079 | t(19)=1.22 , p=0.238 | **t(19)=2.263 , p=0.036** |
